# Critical Review of the Recent Literature on Organic Byproducts in E-Cigarette Aerosol Emissions

**DOI:** 10.3390/toxics10120714

**Published:** 2022-11-22

**Authors:** Sebastien Soulet, Roberto A. Sussman

**Affiliations:** 1 Ingesciences, 2 Chemin des Arestieux, 33610 Cestas, France; 2 Institute of Nuclear Sciences, National Autonomous University of Mexico, Mexico City 04510, Mexico

**Keywords:** e-cigarettes, vaping, aerosol emissions, puffing protocols, organic byproducts

## Abstract

We review the literature on laboratory studies quantifying the production of potentially toxic organic byproducts (carbonyls, carbon monoxide, free radicals and some nontargeted compounds) in e-cigarette (EC) aerosol emissions, focusing on the consistency between their experimental design and a realistic usage of the devices, as determined by the power ranges of an optimal regime fulfilling a thermodynamically efficient process of aerosol generation that avoids overheating and “dry puffs”. The majority of the reviewed studies failed in various degrees to comply with this consistency criterion or supplied insufficient information to verify it. Consequently, most of the experimental outcomes and risk assessments are either partially or totally unreliable and/or of various degrees of questionable relevance to end users. Studies testing the devices under reasonable approximation to realistic conditions detected levels of all organic byproducts that are either negligible or orders of magnitude lower than in tobacco smoke. Our review reinforces the pressing need to update and improve current laboratory standards by an appropriate selection of testing parameters and the logistical incorporation of end users in the experimental design.

## 1. Introduction

Electronic cigarettes (ECs) have become popular substitute products of conventional cigarettes in the framework of tobacco harm reduction, as there is a broad consensus that the aerosol they generate contains far fewer toxic and carcinogenic compounds than tobacco smoke [1,2,3] (see [4] for a diverging opinion). However, users of the devices (“vapers”) are still exposed to the inhalation of harmful or potentially harmful compounds (HPHCs), particularly carbonyls, nitrosamines, metallic compounds and possibly carbon monoxide (CO) and free radicals or Reactive Oxygen Species (ROS). For vaping to fulfill a beneficial harm reduction goal, it is necessary to assess and evaluate laboratory studies that have examined the presence of these HPHC byproducts in vaping emissions.

In a previous paper [5], we reviewed 12 studies targeting metal content in EC emissions. We found that all studies reporting high metal levels (e.g., nickel, lead, chromium and manganese) surpassing toxicological markers suffered from serious methodological shortcomings, especially (but not only) testing high-powered sub-ohm devices at high wattages with the puffing parameters of the CORESTA Method 81 [6,7]) or slight variations of it (i.e., “CORESTA-like”). Almost all laboratory testing is currently conducted by means of these puffing protocols, which were conceived and developed for testing low-powered devices using an airflow rate around 1 L/min and puff volumes below 70 mL. However, these puffing parameters are inadequate for testing sub-ohm devices that require much larger airflow and puff volume to evacuate and condensate efficiently the large amount of vaporized e-liquid produced by the large supplied power. Pending on the wattage range, the combination of high power with low airflow rate and puff volume is either prone or certain to lead to a device testing under overheating conditions. It is also unrepresentative of consumer usage, as sub-ohm devices are mostly used and widely recommended (by manufacturers, vaping magazines and forums) for the ‘direct to lung’ (DTL) vaping style that involves much larger airflows and puff volumes [8,9] (see also [5]).

It is not expected that laboratory testing will reproduce the wide individual diversity (devices, e-liquids, puffing habits) of real-life vaping behavior, but it is necessary and desirable that its experimental setup must be conceived to provide the best possible approximation to the representative characteristics of consumer usage. These facts are recognized by all stakeholders: the official documents of the CORESTA protocol, regulators, academics and consumers (see summary, discussion and references in [10]). A necessary task to evaluate the limitations of the current CORESTA based standard (and suggest upgrades and improvements) is a thorough technical criticism of current laboratory testing largely based on this standard.

To assess current laboratory testing of EC emissions, we apply in the present review a critical analysis of experimental methodology analogous to the one undertaken in [5] but now focusing on laboratory studies detecting nonmetallic byproducts. We provide an extensive review of a literature consisting of 38 articles published since 2018, listed and classified by subject in Table 1 below:

Together with the revision of each individual study cited in Table 1, we also provide an extensive discussion of the physical principles underlying the optimal regime of operation of ECs, the conditions that define representative vaping habits and a summary and evaluation of this literature.

For any given device and e-liquid composition, the appropriate power range for laboratory testing can be determined in the laboratory by an optimal regime characterized by a linear relation between the mass of e-liquid vaporized (MEV) and supplied power W [8]. Underheating occurs below this range with no vaporized e-liquid, while overheating occurs above this range as the relation becomes nonlinear. In the optimal regime, an equilibrium of heat exchange is maintained when a sufficient airflow provides the necessary forced convection (inhalation) to form the aerosol by condensation of the vaporized e-liquid [8,9,49]. Overheating occurs when nucleate boiling gives way to film boiling [22] in which a layer of gas surrounds the coil, propitiating radiative heating exchange, which rapidly increases the rate of vaporization of the e-liquid, breaking the equilibrium sustained by forced convection in the linear regime. While the wick capillarity and e-liquid viscosity decrease as the rate of e-liquid consumption increases with increasingly higher temperatures, the liquid supply to the coil also decreases in parallel with the development of film boiling. The process continues until the coil is dry and thermal energy is radiated (potentially reaching up to 1000 °C) and the wick material (typically cotton) is pyrolyzed at about 450 °C. These conditions produce in end users a burning sensation in the aerosol identified as a “dry puff” or “dry hit”.

The specific power ranges of the optimal regime are device-dependent and can exhibit wide variation in terms of the coil alloys, e-liquid composition and flavors. However, manufacturers provide recommendations of power ranges and usage of the devices and the optimal regime (as described before) provides a laboratory testable procedure to assess these power ranges. When puffing parameters are inappropriate (specially insufficient airflow), these power ranges become narrower, thus facilitating the overheating process even at power settings below the upper limits of manufacturer recommendations. This process can be abrupt in low-powered devices (ciga-likes, pods, second-generation models) whose optimal regime is delimited by narrow wattage ranges. Thus, a small extra supplied power can trigger a rapid onset and development of overheating, especially in devices lacking an inbuilt mechanism preventing this problem. For high-powered devices, the optimal regime occupies a wider range of power settings, so overheating is likely to occur in a more gradual way, making it harder for users to detect it.

Most laboratory testing of emissions has been conducted without carefully monitoring that their experimental setup avoids overheating and unrealistic testing, often (but not always) by testing high-powered devices with insufficient airflow and high power settings above a narrowed power range of the optimal regime. While the emergence of overheating conditions is potentially detectable by sensorial perceptions of users, either by a flavor deterioration or by inhaling an aerosol that becomes too hot, it is well known that users always identify the burning repellent sensation of a “dry puff” that occurs at the end state of an overheating when e-liquid depletes and the wick is pyrolyzed. As shown in a recent study [34], incorporation of end users provides useful guidelines to select the appropriate parameters for realistic and user relevant testing, especially with low-powered devices for which an extra watt can initiate overheating. Unfortunately, few emission studies incorporate input from users in their experimental design. These methodological problems were already identified in the important review by Farsalinos and Gillman [11] of 32 studies on carbonyl byproducts published up to 2017. We find it concerning that five years afterward, these issues still need to be addressed.

The literature on organic byproducts in EC emissions contains detailed and impeccable chemical experiments in reaction pathways associated with the production in the laboratory of these compounds but fail to verify if these chemical processes are plausible or if they are compatible with the physical constraints of the optimal regime. Evidently, an impeccable chemical analysis of EC aerosols might be valuable in itself, but without anchoring its experimental design on the optimal regime, the authors might find results that have little relevance to most end users. The possibility to replicate and reproduce experimental outcomes is a crucially important criterion to evaluate experimental research. Unfortunately, some studies that we revised do not comply with this criterion by failing to disclose sufficient information on important details of their experimental design (puffing parameters, tested devices and e-liquids). Some studies test old devices without providing information on their storage conditions or current state. All this information is relevant to interpret experimental results and possibly replicate them.

Besides comparison between products, evaluation of quality control and fulfillment of regulatory requirements, one of the main tasks of laboratory testing is to assess potential health risks to end users from the presence in EC emissions of potentially toxic byproducts. All studies that we reviewed highlight the toxicity potential of these byproducts, with most studies testing sub-ohm devices concluding serious harm potential to end users from their experimental outcomes. However, our findings in this review suggests that the severity of these risk assessments requires a careful and skeptical evaluation. In some cases, the conclusions of severe risks are questionable, as they emerge from studies that have tested the devices under completely unrealistic and user irrelevant conditions, though in other studies, the risk severity would not apply to the majority of users but only (possibly) to a minority of users with unrepresentative or unsustainable vaping habits.

The section-by-section development of the review is as follows. In Section 2, we explain the physical considerations that define the optimal regime. In Section 3, we discuss various ways to describe and approximate realistic vaping behavior, while in Section 4, we present a methodological description of our review, discussing the conditions of experimental consistency and toxicological realiability as references to evaluate laboratory studies. We summarize in Section 5 two previously published reviews, including a landmark review that examined carbonyl studies published before 2018, providing various key elements of the methodological criticism that we are following in the present review. In Section 6, we review 22 studies focusing on the detection and quantification of carbonyls and other byproducts; in Section 7 and Section 8, studies respectively focusing on CO and ROS; in Section 9, we revise 5 studies whose main focus is the understanding of chemical pathways of byproduct formation from solvent degradation and in Section 10, two studies looking at the relation between carbonyl production and the compensatory behavior associated with low nicotine concentrations. In Section 11, Section 12 and Section 13, we discuss various relevant theoretical issues addressing misunderstandings found in the revised literature, an assessment of risk communication and our conclusions.

## 2. Foreword: The Optimal Regime of Vaping

ECs have been subject to intense scrutiny from their harm reduction role as a substitute product that facilitates smoking cessation. Concerning issues have been raised on their usage among adolescents, impact on health and the pharmacokinetics of nicotine, among many other topics. Unfortunately, there has been little interest in understanding the physical processes that govern the proper functional operation of EC devices.

Since chemical reactions are enhanced by the temperature, many articles have performed temperature measurements of the coil as an attempt to observe the device functionality. However, temperature is an intensive state variable that results from specific conditions that are difficult to control. In the case of a vaping device, the power supplied by the battery heats the wire, transfering heat to the e-liquid and allowing its vaporization into the air induced by the user inhalation. Essentially, the fundamental physical process is heat transfer, and it should then be understood from studying the various involved heat fluxes, especially from the power supplied into the wire surface.

However, few studies have remarked that vaping devices have effective functioning limits: a minimal and a maximal power setting that depends on the wire and device design, the e-liquid composition and also on the airflow rate. These limits are quantifiable by a rigorous relation between the mass of e-liquid vaporized (MEV) vs. supplied power (W). In 2020, Talih et al. [22] published the first article that provides a physical explanation of these limits. Boiling occurs in different form when there is heat flux. Under nucleate boiling (thermal equilibrium between minimal and maximal powers), bubbles are formed on the wire, whereas in film boiling, a local layer of gas surrounds the wire, initiating an efficient process of radiative heat transfer.

**Figure 1:**
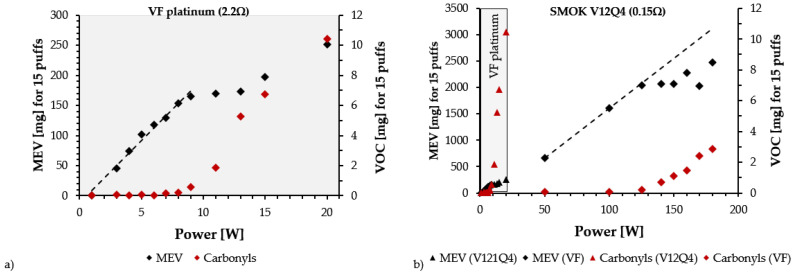
Both panels display vaporized e-liquid mass (MEV) in mg vs. supplied power in W (black squares), with data taken from the experimental results listed in the supplementary file of Talih et al. [22]. Both panels show how in a laboratory setting it is possible to detect the power ranges where the MEV vs. W relation is linear and the appearance of overheating conditions when it becomes nonlinear, coinciding with the onset of an exponential increase of aldehyde production (red squares). The left panel (**a**) shows how for a low-powered device, the optimal regime occupies a narrow power range, while for a high-powered device (right panel (**b**)), the power range is wide. Notice that the power range of the optimal regime of the low-powered device is compressed in the extreme left-hand side of the graph of panel (**b**)).

**Figure 2:**
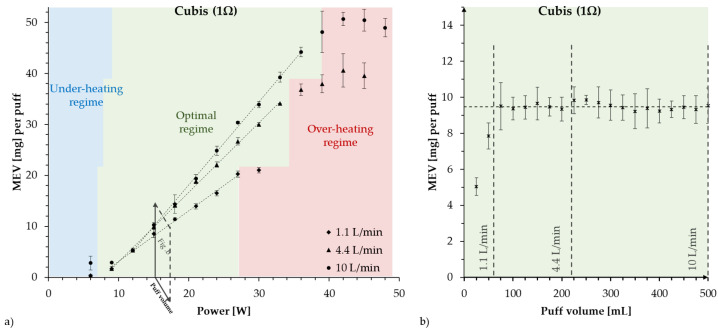
Comparison of two functionality curves for the Cubis 1 Ω device: MEV vs. W for fixed airflow (left panel (**a**)) and MEV vs. puff volume in mL for fixed power at 15 W (right panel (**b**)). Panel (**a**) displays the effects of increasing the fixed airflow from 1.1 L/min (CORESTA conditions), 4.4 L/min to 10 L/min (extracted from [8]). A higher airflow rate increases the slope of the linear relation between MEV and W, allowing for a wider power range under the optimal regime, with appearance of overheating conditions at higher power settings. For low airflow (smaller slope), the optimal regime is limited by a narrow power range. The right panel (**b**) shows MEV to be insensitive to increasing puff volume, apparently suggesting that airflow has no effect on MEV. However, varying airflow under a single fixed power is an artificial testing combination that does not define the optimal regime (see explanation in the text).

Talih et al. [22] also found that the maximal power marking the beginning of the film-boiling regime also marks the starting point of an exponential increase in aldehyde production. Their observations were the same for all tested devices, the two high-powered devices (SMOK TF-N2 0.12 Ohm and V12-Q4 0.15 Ohm) and also for the low-power device (VF platinium 2.2 Ohm). This exponential behavior in reaction rates, also found in articles on CO, is fundamentally linked to Arrhenius relations and reveals a significant temperature increase above the temperatures of optimal conditions. In an optimal regime, e-liquid vaporization occurs under thermal equilibrium or close to it [50]. Therefore, above these equilibrium conditions, e-liquids are overheated in the gas phase by radiative heat transfers. Putting together this knowledge leads us to consider several assumptions based on experimental observations:

- Overheating conditions in which e-liquids undergo temperatures above the boiling temperature of glycerol (VG) leads to significant increase in e-liquid degradation reactions, wick pyrolysis and wire oxidation, leading to a hotter aerosol than that in optimal conditions.
- Overheating conditions are not restricted to high-powered devices, they can also affect low-powered devices. This is illustrated in Figure 1, showing the optimal regime for low- and high-powered devices: a linear relation between mass of e-liquid vaporized (MEV) and supplied power W. The difference is that the optimal regime extends for a wider range of power settings in high-powered devices, while it is restricted to narrow power ranges in low-powered devices. Therefore, it is relatively easy in these devices to enter an overheating condition with little extra supplied power, while for a high-powered device, the deviation from the optimal regime can be more gradual. Several studies have shown that overheating and dry puffs occur in low-powered devices [51].

- The use of a CORESTA regime on devices intended for DTL vaping leads to the narrowing of the power range of the optimal regime range by decreasing its maximal power. This has been confirmed by experimental results reported by Soulet et al. [8] (see Figure 2a below) and Floyd et al. [52]. Therefore, experiments of this type can lead to overheating conditions, even under the power range required by the manufacturer.

- The manufacturer-recommended power range is evaluated before releasing a device into the market, with users identifying a dry puff by perceptions if used above these ranges. Since the boiling point of pure VG (288 °C) and autoignition temperature of an organic material like cotton (350–400 °C) are close, the onset of overheating will initiate cotton degradation, leading to a “dry puff”. In a recent study, which we review in Section 6, Visser et al. [34] matched the chemical characterizations of carbonyls in emissions and the human perceptions of dry puffs in the generated aerosol. Their findings support the claim that dry puff conditions are perceived as a repellent sensation that degrades the pleasant taste of vaping aerosol that prevails under normal conditions [34,53].

A common misconception in several studies (see [20,21,30,38,39,40,41]) comes from testing a device by fixing power W at a single value and varying airflow rate (or puff volume with a fixed puff duration or opening the airflow vents system with fixed puff volume and puff duration). Proceeding in this way necessarily leads to an incomplete account of the involved variables and thus an incorrect characterization of the effects of the airflow. The inhalation induced airflow produces a forced convection on the wire and its influence should be characterized with regards to the supplied heat flux. This fact renders the airflow rate (and more fundamentally air velocity) as a key dynamical parameter that can modify the entire functioning curve of a vaping device (i.e., the minimal and maximal powers and the slope in the MEV vs. W graph of the optimal regime).

As an example of how the effect of airflow can be misunderstood, Figure 2b displays the dependence of the vaporized e-liquid mass MEV on puff volume (i.e., related to the airflow rate for a puff duration fixed at 3 s). Below 100 mL airflow increases MEV, reaching an approximately constant value all the way to 500 mL. This funcional dependence could prompt the conclusion that large airflows (from larger puff volumes at fixed puff duration) bear no influences on the amount of MEV, which any user of the ‘Direct to Lung’ (DTL) style inhaling large puff volumes knows is untrue. In reality, Figure 2a shows that airflow bears a strong influence on the functioning curve, but this must be tested supplying a range of fixed values of W, a single value is not sufficient to characterize the optimal regime. Additionally, we remark that MEV would also have reached a constant value on the same range of tested puff volumes if the experiments depicted in Figure 2 would have been done at other power values, for example, 12 W.

Mathematically, setting the experimental variables as only ‘MEV vs. airflow’ or ‘MEV vs. puff volume’ with a single fixed W results in curves (as in Figure 2b) in a 2-dimensional cross section defined by the plane W constant in the 3-dimensional plot of MEV as a function of W and airflow. Focusing only on a constant W plane obscures the understanding of the role of airflow in balancing the heat transferred by W to condense MEV to form the aerosol under thermodynamically efficient conditions [8]. Experiments fixing W and varying puff volume or airflow might also under (or over) estimate the physical limits of the values that these variables can reach, as these limits are determined by the efficiency of the balance between W, MEV and airflow.

From the arguments presented above, it is clear that experiments with inappropriate air flow will necessary overestimate the risk from exposure to aldehydes over normal conditions. As explained before, the decrease of maximal optimal regime powers due to testing a high-powered device with a low airflow regime (for example 1 L/min) leads to an early onset of the exponential increase of aldehydes that are released and potentially inhaled by the user, but this would not happen in the same power with the proper high intensity air flow (around 10 L/min) used in DTL vaping. Then, a low puff volume instead of a volume consistent for DTL vaping leads to concentration of these quantities in a smaller volume of evacuated e-liquid vapor. This inconsistency between puffing parameters and device power is likely to lead to biased results in a toxicological evaluation, but it does not reflect the representative usage of sub-ohm devices for DTL vaping (see further discussion in Section 3.1, Section 3.2, Section 11 and Section 13).

This section provides the necessary background to understand the main criticism of the studies that we revise in this review. The inconsistency between laboratory testing with a CORESTA or CORESTA-like protocol and majority representative usage of high-powered sub-ohm devices was already mentioned in the review by Farsalinos and Gillman of 2018 [11] of studies on carbonyl byproducts published before 2017. Unfortunately, many recent studies continue testing the devices under these inappropriate conditions, which puts forward the urgency to provide upgraded methodological standards and also to correctly evaluate the consistency of the experiments during the peer review process.

## 3. Realistic Testing vs. Realistic Vaping

Puffing parameters in laboratory testing of EC emissions must mimic as best as possible realistic usage. However, it is necessary to provide robust criteria for what can be understood as “realistic”, since there is a wide diversity of vaping habits. We address this issue in this section.

### 3.1. Vaping Styles: MTL and DTL vs. Device Characteristics

There are two main forms of puffing ECs: the ‘mouth to lung’ (MTL) style (inhalation into the oropharyngeal cavity, momentaneous retention followed by lung inhalation) and ‘direct to lung’ (DTL) style (direct lung inhalation without oral cavity retention). The existence of these two main styles among the diversity in vaping behavior is not an issue of fashion, it has been observed (for example) in studies analyzing videos and films of many vapers in social media (see references in [10]), particularly a large study [54] was able to clearly distinguish the two styles after analyzing 300 videos containing 1200 puffing events from 252 vapers in 14 countries.

Although there is no published demographic evidence directly linking device type with preference of MTL or DTL styles, there are plausible arguments supporting the high compatibility of MTL style with low-powered devices with high resistances and DTL style with high-powered sub-ohm devices. This vaping style vs. device type connection has been long known by retailers, manufacturers and many consumers, with the following arguments put forward and commented in highly trusted vaping forums and magazines [55,56]:

- Low powered devices (ciga-likes, second generation clearomizers, cartridge and refillable pods, tank stating kits) typically operate at powers well below 20–25 W, have narrow mouthpieces and thus involve lower puffing volumes under high air resistance, similar to smoking. Beginner vapers (many of whom are still current smokers) tend to adopt the MTL style that is close to the puffing habits and puff volumes of cigarette smoking [57,58]. Typically, vapers using low powered devices for MTL style use PG dominated e-liquid solutions with higher nicotine concentrations.
- High powered sub-ohm devices operating at W > 40–50 W, use external batteries, often allow users to set up power/temperature, are more bulky and expensive than low powered devices, thus requiring higher level of maintenance and expertise. Their mouthpieces are wide and thus their low air resistance facilitates drawing large puffing volumes that need not be retained in the reduced volume of the oropharyngeal cavity. Therefore, they are not likely preferred by beginners or vapers simply trying to quit and remain smoke-free, but by veteran vapers who have quit smoking long ago and thus tend to enjoy the recreational hobby-like aspect of vaping, often puffing large clouds, using low nicotine concentrations and e-liquids with predominantly VG content.

Evidently, the rapid development of vaping technology and the growth and diversity of the vaping market have introduced a continuum of device types, including those of intermediate power (20–40 W) compatible with both MTL and DTL style and sub-ohm and supra-ohm resistances. with many vapers gradually becoming able to practice both styles: DTL with a sub-ohm device in situations in which emission of large aerosol clouds is not disturbing to bystanders (at home) and MTL when they need to vape discretely. Vapers gradually learn to follow their sensorial faculties to adapt to the diversity of devices according to their personal needs, though naive beginners or smokers trying to vape may experience unpleasant extreme situations when puffing a given device with the wrong ‘technique’ (see candid descriptions in [55]). Unfortunately, there is still insufficient published demographic data to assess vaping behavior.

In spite of the increasing diversity of vaping behavior, consumer magazines and forums comment that the two main vaping styles and their connection with device types still remain roughly well defined and distinguishable. These anecdotal accounts agree, in general, with available observations in studies cited by [10]. It is also consistent with the footage material examined in [54]: 80% of users of sub-ohm devices practice DTL and 98% of DTL vapers use sub-ohm devices, while 95% of users of low-powered supra-ohm devices practice MTL. However, the rapidly evolving dynamics of the vaping market might lead to substantial changes in the prevalence of these styles, such as a gradual increase of consumer preference for new low powered pod devices in the US [59], the UK [60] and Germany [61], as well as increasing popularity of low powered disposable devices, specially among young adults and teenagers [60,62,63].

The connection between vaping style and device type is relevant to assess emission studies, most of which have been carried on with CORESTA or CORESTA-like protocols, which should be appropriate for testing low powered devices used with low airflows and puff volumes comparable to cigarette smoking. However, as we argued in Section 2, testing high powered sub-ohm devices with the low airflows and puff volumes used by these protocols can be very problematic, as it increases the likelihood of overheating by narrowing the power range of optimal regime (approaching overheating conditions might be detected as a flavor deterioration, see Section 3.3). CORESTA or CORESTA-like protocols might be inconsistent with the majority consumer usage of sub-ohm devices for DTL vaping that involves large airflows (see Section 3.2). As we show in Section 6, Section 7, Section 8, Section 9 and Section 10 and summarize in Section 11, at least half of all revised laboratory emission studies have tested sub-ohm devices with CORESTA or CORESTA-like protocols. We discuss the shortcomings of this testing in Section 12 and the implications for health risk assessment in Section 13.

### 3.2. Inhalation Behavior

Inhaling through an EC device involves overcoming a specific pressure drop that must be added to the pressure of rest breathing a tidal volume around 500 mL, roughly 10% of vital capacity, with deeper inhalation involving more exerted pressure and inhaled volume. Every device has a specific air resistance coefficient linking the pressure drop generated to the airflow rate passing through. The physiological limits of full vital capacity (10 kPa) and this device-specific air resistance determine the physiological range of airflow rate in real life vaping. The volume capacity of the oropharyngeal cavity (100–170 cm^3^ [64]) places physical limits to the amount of air diluted aerosol that can be puffed and flushed with a given puff duration for the mouth retention in MTL vaping, while no such limit occurs in DTL vaping.

Devices used for MTL vaping are mainly designed with small air inlet holes (diameters around 1–2 mm), which leads to a high air resistance that significantly reduces the range of possible airflows. As an example [49], the Eroll device from Joyetech allows an airflow range of 0–2.8 L/min, with the user inhales very small volume even at the top value 2.8 L/min. Assuming a middle value of this range at 1.4 L/min and a puff with the rest tidal inhalation volume of 500 would imply a puff duration above 20 s, a long duration that is not only unrepresentative, but uncomfortable (normal breath lasts less than 5 s). Therefore, a user necessarily has to inhale a lesser aerosol volume that dilutes in air.

As a contrast, devices meant for DTL vaping have larger air inlet holes or a groove, all of which significantly reduces their air resistances, even leading to negligible values. Therefore, the user will be able to generate significantly higher airflow rate. As an example, in the tests conducted in [49] most of the DTL devices only reached 20% of the maximal pressure of the lung at at airflow of 10 L/min, thus suggesting that the airflow rate can be, at least theoretically, significantly higher. Average adults under rest conditions breath 12–16 inhalations per minute, with breaths lasting on average 3.75–5 s and airflow rates of 7.5–10 L/min, with both frequency and volume increasing with effort.

However, vaping is mostly a recreational activity that tends to occur at an intensity close to resting conditions, so we can assume that, given the low to negligible air resistance of sub-ohm devices, DTL vaping involves inhalation through puffs whose volume will be close to that of the average resting tidal volume (500 mL), as the body does not require an extra consumption of oxygen. A “cloud chasing” competition or a presentation of a device might trigger an extra “performing” effort involving higher puff volume, but these are infrequent extreme situations. Additionally, given the extra effort needed to overcome the pressure drop of the device, we can also assume that puff duration will tend to be shorter than in a resting inhalation. While we do recognize the wide variation in puffing habits (even within DTL and MTL styles), we believe that a puff volume of 500 mL and puff duration of 3 s, with a resulting airflow rate of 10 L/min, seems to be appropriate to characterize on average DTL as a plausible hypothesis that needs to be tested experimentally.

### 3.3. Organoleptic Perceptions

Flavouring compounds emulating fruity, mint, tobacco and sweet desert tastes [65] are essential for e-liquids solutions to generate a pleasant aroma/taste sensation during vaping. The deterioration of these sensorial experiences can also signal users that their device might not be functioning normally. Under optimal conditions e-liquids are heated at temperatures below the boiling temperature of pure glycerol VG (288 °C), with heating elements still wet, at least close to the e-liquid, with the porous structure in the wick also wetted. Passing towards overheating conditions leads to local drying that increases with increasing power. The porous structures are built up with a sheet of cotton, mainly composed (>90%) of cellulose, which is a biopolymer made of a linear chain of D-glucose. Since the 1980s, the wood industry has undertaken well documented studies of cellulose pyrolysis through a heating process from 20 °C to 800 °C. This pyrolysis is not uniform and can be separated in four main stages [66,67]:

- Below 100 °C, cellulose loses water that is contained in its fibers.
- Between 150 °C and 290 °C, dehydration reactions occur resulting in a small weight lost.
- Between 290 °C and 380 °C, fast depolymerisation of cellulose happens releasing close to 80% of volatile condensable compounds (boil-oil) as levoglucosan reaching 60%, furans as 5-hydroxymethylfurfural (5-HMF), 5-methylfurfural (5-MF), furfural, furfuryl alcohol and gaseous compounds as CO, CO${}_{2}$ and small chain compounds (glycolaldehyde, acetaldehyde, acetol).
- Between 380 °C and 800 °C, boil-oil also contains phenols and ketones compounds formed by the charring process of the remaining solid structure, with an important release of methane and CO resulting in a carbon mass at 800 °C.

An extensive discussion of the different processes during cellulose pyrolysis can be found in the review of Collard and Blin [68]. Bearing in mind the pyrolysis process outlined above, is is not outlandish to assume that the onset of overheating conditions in vaping can easily initiate the fastest stages of cellulose pyrolysis, resulting in additional mass loss that can be measured during the generation of emissions. Under such conditions, extra condensable and non-condensable compounds might be added to the gas phase of the aerosol generated by the vaporization of the e-liquid. Some of these new molecules are furans, like furfural and 2-furanmethanol, producing bread/burnt type of odors and a bitter taste. These organoleptic properties of condensable compounds are also well documented by the food industry, which uses wood burning to provide some specific flavours to various food items: smoked fish, meat, cheeses and other food items [69,70]. Additionally, some polycyclic aromatic hydrocarbons (PAHs), classified as respiratory HPHCs, are also produced mainly at temperatures above 400 °C [71].

Although the physicochemical processes linking the dry puff phenomenon to the cotton pyrolysis process have not been well researched, there is solid evidence documented in the literature of cellulose pyrolysis and wood burning, processes that release new chemicals that affect sensorial perception. While there is no experimental evidence that these effects are noticeable in vaping, it is not far fetched to assume that earlier stages of an overheating regime (temperatures around 280–300 °C) could trigger the early stages of this process through a deterioration of flavorings due to the extra release of chemicals. However, MTL devices have small heating coil surfaces and the passage from the optimal regime to overheating might occur more abruptly in more compressed power ranges (see Figure 1), but this passage can be gradual enough in DTL devices designed with significantly larger coil surfaces and normally used with large airflows (which favors lowering temperatures). Therefore, dry-puff conditions can be perceived as a local abrupt event in low power devices, but in DTL it should be sufficiently gradual to be perceived as a kind of taste deterioration by users.

### 3.4. Puffing Frequency and Duration

Given the advance in EC technology, it is not surprising to find a wide variation in puffing habits among the millions of vapers worldwide. Evidently, the standardized and regimented laboratory testing parameters will never provide a precise fit to real life vaping, but to evaluate how good an approximation it can be, it is unavoidable to consider averages and representative puffing parameters obtained in observational studies of vapers under natural conditions, with an understanding of their scope and limitations.

Early original observational studies of vaping habits (see review by Prasad [72]) showed similar mean numbers of daily puffs (mean ± SD): 225±59 in [73], 163±138, median = 132 in [74] and 78±162 in [75], though reporting an enormous variability in the full range of daily puffs, for example: 24–1091 in [73] and 1–1286 in [74]. Vapers in these studies used first and second generation devices whose nicotine delivery rate was inefficient. Subjects in more recent studies [47,76,77] used second and third generation devices that allowed modifying power setups and with better nicotine deliver, thus reporting a compensatoty effect with more daily puffs for lower nicotine levels (see Section 10). These studies also report higher numbers of daily puffs, for example, we have from [47] (mean ± SD): 338±161 (low nicotine level fixed power), 308±135 (low nicotine level variable power), 279±127 (high nicotine level fixed power), 272±128 (high nicotine level variable power), where low and high nicotine level respecively given by 6 mg/mL and 18 mg/mL.

Besides counting daily puffs, it is important to remark that real life vaping follows circadian patterns that are not regimented. As shown in [74,78] puffs numbers cluster at daily hours of wakefulness (8 h to 23 h), identifying certain usage patterns: relatively regular puffing with large interpuff separation, short periods of very frequent short duration puffs (likely reminiscent of “cigarette breaks”) and longer periods with long duration infrequent puffs (these patterns are also supported by videos of vapers [54]).

Considering the puffing data that emerges from these observational studies, we believe that the upper range of daily puff numbers (over 1000) found in [73,74] are unrepresentative outliers that can be ruled out. Assuming a wakefulness time of 16 h, 1000 puffs imply puffing every minute 1000 times, an excessive regime, more so considering the fact that vaping is not allowed in most work places, which significantly reduces the circadian period when vaping is possible.

Given the daily puffs outcomes from more recent studies and considering circadian variation comprising periods of frequent and infrequent puffing, we believe that a useful mean value that is most representative of vaping usage is 250–300 daily puffs, obviously understood as an estimator that roughly incorporates daily variation. This value is useful as a criterion to evaluate qualitatively how much interpuff lapses and puff duration in laboratory testing can be a reasonable approximation to observed patterns in a daily time frame. It is also useful to evaluate daily exposure doses given experimental outcomes expressed as a “per puff” basis. Thus, it is misleading to extrapolate to realistic vaping a study involving 50, 100 or 300 regimented puffs with a 10 s interpuff interval, as this frequent puffing has been observed to occur in a short timeframe involving 10–20 puffs, but it is not representative of daily behavior. Likewise, we can regard as unrealistic very long puff duration times, even if reported in observational studies (5.6 s is reported as 95% percentyl in [54]). Rather, we will consider as representative values 2–4 s puff duration.

## 4. Methodology: Towards a Consistency Standard of Laboratory Testing

**Figure 3:**
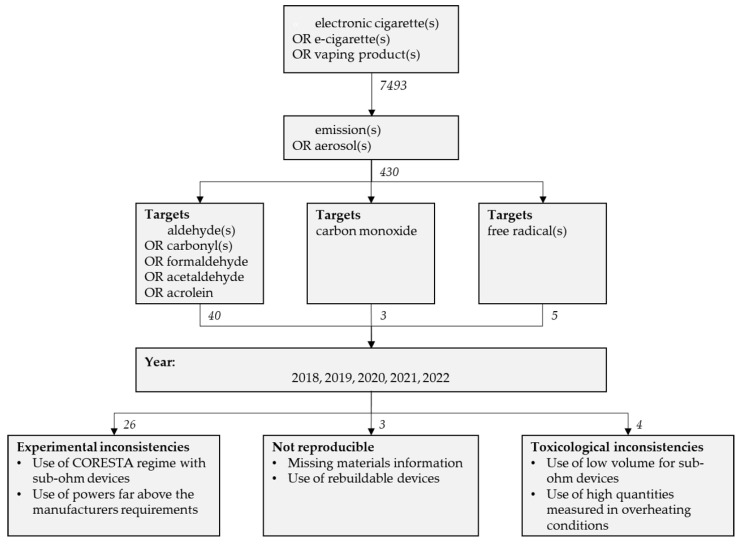
Methodological algorithm used to selecte the papers of this review.

This extensive review focuses on published articles on emissions form vaping products, with experiments aimed at characterizing byproducts generated by EC aerosol. We used the PubMed database applying the algorithm illustrated in Figure 3 with the following terms:

- electronic cigarette(s) OR e-cigarette(s) OR vaping product(s)
- AND aerosol(s) OR emission(s)
- AND (aldehyde(s) OR carbonyl(s) OR formaldehyde OR acetaldehyde OR acrolein OR acetone OR crotonaldehyde) OR (carbon monoxide) OR (free radical(s))

Since the review by Farsalinos and Gillman [11] has already revised studies published up to 2017, we excluded studies published before January 2018. From this searching process we found the 38 articles cited and listed in Table 1, mostly dealing with aldehydes, but also CO and and free radicals. We will evaluate these articles under the following criteria which we believe can provide a useful guideline for improving the quality of laboratory testing of EC emissions:

1. **Experimental consistency.** The consistency between the experimental procedure (puffing parameters, devices, analytic methods) and the best approximation available to user behavior. Experimental inconsistencies occur mainly between the type of device tested and (i) the puffing protocol, (ii) a supplied of power higher than the limits recommended by the manufacturer as inferred from the optimal regime.
2. **Reproducibility of the experiments**. The articles under revision must provide sufficient information that allows, in principle, a possible replication of the experiments. Vaping aerosol requires for its generation the usage of: a device (mod and atomizer), an e-liquid and a vaping regime (puffing). The authors must also provide the commercial name of the devices, as well as the technical information on the coil used (if it is a removable part), the commercial name of the e-liquid with as full information as possible, including the e-liquid composition if it is an in-lab production, all this together with the vaping regime: puff duration, the airflow rate, puff frequency, number of puffs/series. Experiments conducted with rebuilt devices (“Do It Yourself” devices) cannot be considered relevant to approximate real usage, as they are handmade coils.
3. **Toxicological confidence**. The authors must provide detailed account of the experimental outcomes to correctly compute daily exposure (with the right time frame and air dilution volume) and compare it with toxicological threshold limits published by official organizations. The utility and relevance of this comparison is closely tied to how well the study complies with the criteria of Experimental Consistency and Reproducibility, otherwise the risk assessment is either an over (or under) estimation, speculative, irrelevant to end users or only applicable/relevant to special minority niches.
4. **Old and/or used devices**. Authors testing such devices must communicate their storage conditions and current state, as well as justify the reason why such devices are tested. This is important, as there is evidence that devices older than 2–3 years (used or new) may degrade and undergo leaking corrosion (see full discussion of this issue in [79]).

We provide in Section 11 a color/symbol code system (tick marks and traffic lights) to evaluate how well the studies we review in the following sections comply with these quality criteria.

## 5. Previously Published Review Articles

### 5.1. A Groundbreaking Review

Farsalinos and Gillman published [11] in 2018 an important review of 32 studies obtained by a PubMed search of studies published between 2013 and 2017 focusing on carbonyl byproducts. The authors comment on the wide (ad hoc) diversity experimental procedures, producing an enormous range of outcomes in concentrations, all of which is symptomatic of a lack of a consensual testing standard in this extensive literature, thus hampering the possibility of an objective comparison and interpretation of obtained results. While the overwhelming majority of the 32 reviewed studies tested the low-powered devices that were of common usage before 2017 (first and second generation ciga-likes and clearomizers), all of which are today either obsolete or of marginal use, the methodological critique by Farsalinos and Gillman is still relevant in assessing present day lack of proper standards in laboratory testing. As we argue in Section 2, Section 3 and Section 4 (and in our recent review of metal studies [5]), unrealistic testing, overheating and possible dry hits can also occur in emission studies testing devices available today (specially, but not only, third generation sub-ohm models).

In discussing the methodological considerations, the authors remark that excessively large concentrations of carbonyl byproducts (specially, but not only, formaldehyde) detected in some studies might be a consequence of machine testing the devices under inappropriate and/or unrealistic conditions, such as: puffing parameters that bear no connection with real life habits of consumers: too short inter-puff lapses and/or excessively long duration puffs, low puff volumes for third generation tank devices that were available commercially at the time of the review (2017). The authors place special emphasis on the specific “dry puff” phenomenon, a terminal overheating condition that arises as the e-liquid in the atomizer depletes and the supplied power pyrolyzes the wick (see Section 2 and Section 3.3), all this taking place while vaping machines continue operating. The authors emphasize that only 4 of the 32 reviewed articles explicitly verified absence of a dry puff during testing. They argue that the simple ad hoc assumptions made by most authors on puffing parameters or ranges of supplied power would be insufficient to prevent or identify a dry puff during the experiments, but actual vapers participating in the testing can easily identify it by its “organoleptic” or sensory effects (a burning repellent taste). However, as we show in Section 2, the authors’ claim that dry puffs dramatically increase carbonyl production is not speculative, but a fact verified experimentally, which complements sensorial testing by actual vapers as in [34].

Farsalinos and colleagues conducted in 2015 an observational laboratory experiment, aided by voluntary vapers, showing that excessively high aldehyde production only occurs under dry puff conditions [80]. However, they published replication studies (summarized in [11]) to reproduce the outcomes of two laboratory studies, Jensen et al. [81] and Sleiman et al. [82], which generated controversy and alarmist media headlines by detecting extremely large aldehyde concentrations much higher than in cigarette smoke:

- The experiment by Jensen et al. [81], published as a letter to the editor, examined the emissions of a top coil C4 device (now obsolete), detecting extremely high levels of formaldehyde hemiacetals, not formaldehyde, yet using the outcomes of their tests they estimated a lifetime cancer risk for formaldehyde inhalation in vaping that was 15 times higher than that from estimates from cigarette smoking. The replication in [83] showed using the same device and e-liquid that such levels only occur for abnormal usage under overheating and human identifiable dry puffs. Given the implications and the widespread diffusion of [81], there were calls for its retraction [84]. The team of Jensen et al. responded to this criticism in this communication [85], but as stated in [86], they could not deny that these worrying levels of byproducts did happen under abnormal usage conditions.
- Sleiman et al. [82] tested two devices: a top-coil and a bottom-coil, both with a silica wick atomizer filled with a commercial tobacco-flavored liquid. High levels of carbonyls were found (in ng/mg): formaldehyde 1300–48,200, acetaldehyde 260–19,080, acrolein 120–10,060, acetone 70–1410 and crotonaldehyde 10–720, with levels in the upper end of the ranges far exceeding the respective emissions from tobacco cigarettes, leading to warnings about serious risks from vaping. For the replication in [87] Farsalinos and colleagues used the same devices and aided by volunteering vapers showed that such high emissions happened under clear dry puff conditions, with usage in the normal ranges producing outcomes closer to the low end of the ng/mg ranges well below respective levels in cigarette smoke. They also tested a more recent device mentioned in [82] finding even lower aldehyde levels.

Another study by Khlystev and Sambureva [88], also reviewed in [11], claimed that flavorings by themselves generically dominate carbonyl production and lead to excessively large aldehyde production in comparison with testing the same device with nonflavored e-liquids. This study was also replicated in a comment by Farsalinos and Voudris [89], using the same device and (what seemed to be) the same e-liquids, showing that flavorings did not produce this large enhancement of aldehydes. This lead to an interesting exchange with Khlystev and Samburova replying to the comment [90] and Farsalinos and Voudris countering that reply [91]. In a more recent study in 2020 Gillman et al. [92] found that flavorings do contribute to aldehyde abundance, but at much lower levels of enhancement than those reported in [88].

### 5.2. A Recent Descriptive Review

Ward et al. (2020) [12] present a descriptive review summarizing the extensive literature on potentially harmful chemicals in ECs emissions, commenting very briefly only on the main outcomes. Toxicants are classified in 6 major categories: carbonyls, volatile organic chemicals, trace elements (metals), reactive oxygen species and free radicals, polycyclic aromatic hydrocarbons and tobacco-specific nitrosamines. The review contains 92 articles selected through the PRISMA search protocol. All studies we are revising in the present review (and in our review on metals in [5]) are also cited by Ward et al.

The review is a valuable source for references, but the reviewed articles are barely described without any critique. Given the large amount of cited studies, a merely descriptive approach is understandable and justified, though readers should be aware that substantial information is missing when presenting outcomes of studies without evaluation. To illustrate this point, we use an example taken from the section Trace elements of Ward et al, who cite a study by Ting et al. [93] (their reference 54) mentioning that it “identified that 5% of ECs and e-liquid combinations tested emitted Cr at levels that exceeded permissible daily exposure limits”. However, the study of Ting et al. shows several serious flaws, making their outcomes completely unreliable: they did not identify the tested ECs and characteristics (brands, coil resistance, power/voltage ranges), did not specify the power levels in which the devices were tested and assumed an unrealistic amount of 1200 daily puffs to compute exposures. Evidently, this study has no utility or relevance to end users, yet readers will assume that it has the same quality as other cited studies.

## 6. Studies Focused on Quantifying Carbonyls and Other Byproducts

We present in this section an extensive revision of 22 studies mostly focused on laboratory experiments to quantify the presence of carbonyl byproducts in EC emissions, though some of the studies also discuss in detail pathways of thermal degradation for specific compounds. We exclude studies published before 2018, most of which were reviewed by Farsalinos and Gillman [11].

### 6.1. Studies Published in 2018

**Vreeke, Peyton and Strogin**. The authors [13] used NMR spectroscopy to examine the role of the chemical Triacetin (TA, used in “do it yourself” and in commercial e-liquids) in the enhancement of the production PG and VG degradation aldehydes (formaldehyde hemiacetals, acrolein and acetaldehyde), to explore the possibility that this enhancement might be derived directly from the flavoring molecules. They tested a sub-ohm device SMOK Alien 220 W with a SMOK Baby 0.4 Ω single vertical coil (at 55 and 65 W) and with a Kanger Protank 2 Clearomizer with a 2.2 Ω single horizontal coil (at 9 and 11 W), using a CORESTA protocol: 3 s puff, 30 s interpuff period and a 55 mL puffing volumes. Compared with an e-liquid with a 50:50 P/G mix and no TA, both devices with e-liquid containing 10% TA produced a significant increase in aldehyde levels, of up to 185% (about twice as much). Noticeably, save for the device with 2.2 Ω at 11 W, acrolein and acetaldehyde were only detected when TA was present in the e-liquid and the enhancement was larger for that device.

Information about aldehyde enhancement from specific flavoring chemicals is certainly useful for e-liquid manufacturers, regulators and consumers. The enhancement of aldehyde production was found in this study for the sub-ohm device at 55 and 65 W was. While these power levels might be within manufacturers recommendations when the device is used with airflows of DTL vaping, they should be in the overheating region when puffed with a reduced CORESTA airflow that narrows the power ranges of the optimal regime. Hence, these experimental outcomes were very likely obtained under overheating conditions that are either unrealistic or only relevant for a minority niche of users (see Section 2, Section 3.1 and Section 12.1). The other combination of a powerful mod battery with an outdated clearomizer is very odd, as users would normally attach this clearomizers to low-powered mods.

**Korzun et al.** The authors [14] examined the effect of flow rates and levels of specific aerosol toxicants produced in EC emissions, arguing that their wide variation among users can be a confounding element in aerosol chemistry. Large airflows can lower coil temperatures and thus decrease toxicant production, a relevant fact for users of sub-ohm devices doing the DTL vaping style that involves large airflows and puff volumes (to generate “large clouds”). The authors argue that large airflows do not favor intermediate aldehyde formation compounds along the chemical paths of PG and VG degradation. However, the authors’ choice of the largest airflow in their experiments, 36 mL/s (roughly 2 L/min), is far below the typical airflows used in DTL vaping (about 170 mL/s or about 10 L/min [8]).

The tested device was a Tesla Invader III with a KangerTech SubTank Mini atomizer with operational power of 15–30 W, single bottom coils with a 1.26 Ω and 50:50 PG/VG e-liquids, tested at 11, 13, 17 and 24 W. Sessions of 20 puffs with two protocols: CORESTA (3 s puff duration, 30 s puff interval, 55 mL puff volume) and two Custom Square Mode (3 s puff duration, 30 s puff interval, 21 and 198 mL puff volume), flow rates of 18.3, 7.0 and 36 mL/s, respectively. Analytic determination by quantitative NMR (qNMR).

While testing at 11–17 W showed toxicants with concentrations below LOQ and LOD, the authors argue that the main hazard to end users is the excessive exposure to the solvents, particularly PG. The tested device at 24 W and 18.3 ml/s emits 18.5 mg/puff of PG/VG, assuming a 50:50 PG/VG partition as in the e-liquid and 25 puffs/h leads to 115.6 mg/h of inhaled PG. The authors compare this value with 75 mg/h inhaled PG, the dose from the 1 h inhalation threshold of the Spacecraft Maximum Acceptable Concentration of the NASA, concluding that PG inhalation poses a serious harm to users. This risk assessment is highly questionable, as the cited SMAC document warns that its threshold values are only applicable to a spacecraft environment (an extremely enclosed and isolated environment that bears no relation with real life vaping scenarios). Using a more reliable source looking at respiratory effects of PG aerosol inhalation in healthy human subjects [94], mild exposure related symptoms only occur at 871 mg/$m^{3}$ which (for 20 $m^{3}$ daily air breathing by adults) implies an inhalation of 725 mg/h of PG, 7 times above the inhaled PG from the tested device.

**El Hellani et al.** This study [15] assesses nicotine and carbonyl yields in popular low-powered devices in the U.S. market as of 2017: they tested 27 devices, disposables, pr-filled cartridges and tank models of 10 brands. E-liquids were in three flavors: tobacco and menthol a third different one, with 7.11–20.90 mg/mL nicotine concentration and a wide variation of VG/PG ratios and power ranges around a 5 W average. Sessions of 15 puffs were conducted, with puffing parameters selected to “represent an experienced user”: 4 s puff duration and 10 s inter-puff duration with a puff velocity of 1.5 L/min. However, this excessively high puffing frequency is unrepresentative of EC users (though it may be representative of smoking breaks of cigarette smokers). It also increases the possibility of high carbonyl yields and even dry puffs [11,16].

In total 12 carbonyls in the gas phase were targeted for HPLC analysis, including formaldehyde, acetaldehyde, acetone, acrolein. However, the way the authors report and compare carbonyl yields and concentrations is misleading. They report a range 3.72–48.85 μg/15 puffs of carbonyl yields (also in the abstract), without mentioning that (from their Table 2 and Figure 2), the high end value of this range corresponds to only two unrepresentative outlier values (in 27 measurements) with 24 quantifications below 11 μg/15 puffs. As the authors recognize, such outliers are necessarily produced by dry puffs, and thus denote abnormal usage. They report formaldehyde concentrations of 0.58–5.05 mg/$m^{3}$, again without mention that the high end of this range corresponds to the same 2 unrepresentative outliers. These concentrations are lower than those in tobacco smoke (4.6–148.9 mg/$m^{3}$), but above measured human breath (<0.5 mg/$m^{3}$) and the short term 15 min exposure limit (REL of NIOSH) 0.123 mg/$m^{3}$. However, comparison with formaldehyde in human breath is irrelevant to assess exposures from ECs and the right occupational marker to compare EC emissions is not the 15 min SREL-NIOSH, but the PEL-NIOSH 0.92 mg/$m^{3}$ (or 0.75 ppm) for time averaged 8 h lifetime exposure, which is above the representative quantified formaldehyde concentrations around 0.6 mg/$m^{3}$.

### 6.2. Studies Published in 2019

**Beauval et al.** The authors [16] show that, together with multiple other factors (power, temperature, device architecture, e-liquid composition, flavorings), the choice of puffing parameters (specially puff duration and interpuff frequency) significantly influences aerosol yields and outcomes of aldehydes, a fact that complicates an objective comparison between studies and interpretation of their results. They also provide a summary of reported concentrations (in ng/puff) of formaldehyde, acetaldehyde, acetone, acrolein, propionaldehyde and methyl-glyoxal, reported in 20 studies published between 2013 and 2017 (all of which were reviewed by Farsalinos and Gillman).

Aerosol was generated from a single e-liquid (PG:VG 65/35, mint flavour; 16 mg/mL nicotine) by operating two devices from the French manufacturer NHOSS: a second generation “Lounge” model, 2.8 Ω nichrome top-coil, 4.6 W and a third generation “Mod box TC” model with Air Tank claromiser, 0.5 Ω kanthal bottom-coil and power supply 7–50 W, tested at ranges recommended by the manufacturer 18–30 W. They used DNPH cartridges for carbonyl collection and HPLC-UV for analysis. Aerosol temperature at the mouthpiece was measured by a NTC 3950 thermistor in separate tests under same conditions. Overheating and dry puff were controlled by monitoring also e-liquid consumption and replacing atomizers after each 20 puff session. Environmental contamination controlled by blank collections before each experiment.

Seven puffing regimes were defined for the tests, the standard CORESTA regime (puff volume: 55 mL, puff duration 3 s, puff frequency: a puff every 30 s) by the following modified parameters: puff volumes 35 and 100 mL (PV− and PV+, airflow 0.21 and 0.6 L/min), puff duration: 2 and 6 s (PD− and PD+) and puff frequency: 1 puff every 60 and 14 s (PF+ and PF−). The alteration of EC components after 480 puffs was also considered (the initial and final CORESTA, IC AND FC).

For the Lounge and TC 18 W consumed e-liquid was 5–10 mg/puff, with largest values produced by longer puff PD+, while the TC 30 W consumed 15–25 mg/puff with largest values produced by larger puff volume PV+. Regarding carbonyl outcomes, if we remove unrealistic PD+ and TC 30 W (a sub-ohm device tested with a low airflow) then concentrations are negligible (well below 1 μg/puff). Concentrations ranges (ng/puff): 20–255 (formaldehyde), 29–364 (acetaldehyde), 4.4–28 (Acetone), ND-40 (acroleine), 1.0–32 (propionaldehyde) and 4.5–141 (methyl-glyoxal).

The measured temperatures at the mouthpiece show smooth logarithmic-like curves that increase during the fist 5 puffs and reach a sort of plateau. These temperature measurements were not validated and are not reliable, since aerosol temperature time variation must be sensitive to the puffing sequence, producing saw teeth profiles. Nevertheless, it is interesting to qualitatively compare the temperature curves between the different graphs, as all were obtained with the same instrument and method. This comparison shows higher plateau temperatures for longer and more frequent puffs (PD+ and PF+) for both tested devices and all power settings. Also, the largest plateau temperatures occurs for the TC 30 W, which provides qualitative support to the inadequacy of testing a sub-ohm tank model at its higher power level with a low flow rate of at most 0.6 L/min.

**Ooi et al.** The authors [17] first sample emissions with a device made of an Innokin Iclear 30 S (Shenzhen, China) atomizer with a Kanthal coil and an Istick 30 W battery with a variable voltage of 2.0–8.0 V (no information is provided on power levels and coil resistance). E-liquids with various VG/PG ratios were used for GC-MS analysis, with the E-cig was operated at 4.80 V and vaped at 3 s per puff for a total of 12 puffs, with 30 s interpuff lapse and airflow 2.0 L/min (they refer their puffing parameters to their reference [14] published in 2014, which did not not use this airflow). The authors only report increasing presence (through GC-MS spectra) of carbonyls in aerosol emissions from e-liquids with a higher VG/PG ratio, but do not quantify the analytes, reporting only carbonyl outcomes from old studies published between 2010 and 2014.

After describing the limitations of the GC-MS analytic technique (analyte condensation inside sampling bags absorption into the aerosol phase prior to sample analysis), the authors sample and analyze the vapor phase by Fourier Transform Infrared Spectrometry (FTIR) in the emissions of a sub-ohm device Joyetech eVic-VT E-cig device with variable temperature control (ranges 200–600 F), at two temperatures: 500 F (260 °C) and 600 F (316 °C). No information is supplied on the puffing protocol. Emissions were generated for immediate analysis by FTIR, and thus (according to the authors) the obtained concentrations were much higher than the nondisclosed ones obtained with GC-MS and the other device: 1236±361 mg/$m^{3}$ at 260 °C and 3250±449 mg/$m^{3}$ at 315 °C, as well as 8.91±0.07 mg/$m^{3}$ for CO at 315. However, these concentrations are meaningless without knowing the puffing parameters used for the Joyetech eVic-VT, a sub-ohm device that can run to high power up to 230 W and that they tested at its maximal temperature. The authors remark that their CO emissions were lower than those reported by El Hellani et al. [36] which surpass National Ambient Air Quality Standard, but (as we show in our comments on that study in Section 7) these outcomes correspond to unrealistic puffing parameters and thus are irrelevant for end users.

**Balushkin et al.** This comprehensive study [18] was funded by Philip Morris International. Thirty-four samples were tested of commercial devices purchased in 2015, 2017, and 2018: closed disposable “cigalike”, cartridge systems and open tanks models (brands listed in supplementary file) and analyzed with 57 e-liquids brands and flavors, together with an internally prepared reference e-liquid (39.1% PG, 39.1% G, 1.8% nicotine, and 20% water) used in testing open tank systems.

The authors focus on carbonyls, specially: acetaldehyde, acrolein, and formaldehyde, though other HPHCs (nitrosamines, metals) were also targeted and generally found not quantifiable. The devices were tested from maximal e-liquid levels until full depletion, in horizontal position and at the highest temperature or power setting (for devices with variable temperature or power). Carbonyl compounds were analyzed using HPLC-UV. The study follows the CORESTA method 81 standardized aerosol generation and collection protocol, though slight variations of this protocol were used only for 9 closed systems, but these small puffing protocol deviations had little effect in their carbonyl emissions.

The authors define the “end of life” criterion for e-liquid depletion (12.5 mg mass loss per blocks of 50 puffs) to allow for a direct comparison of products and avoidance of dry puffs. Outcomes are reported on a per-puff basis computed from the lifetime average yields. The study shows that generally low-powered closed systems produce the lowest levels of lifetime average yields of carbonyl emissions (18.9–10,700, formaldehyde (see their Table 3), while these emissions are in general higher in open tank systems (538–53,400 ng/puff, formaldehyde (see their Table 4). However, these outcomes might be overestimations with respect to real life usage, as users might feel a foul flavor well before high lifetime percentages arise. Also, some of the tested devices were acquired as far back as 2015, which does not rule out corrosion and leaching effects, given the lack of information on their storage conditions.

Some of the results of the study provide very useful information to consumers. As shown in the examples displayed in Figures 4 and 5 of Baluskin et al., outcomes of formaldehyde in a closed system increase by an order of magnitud as the device reaches 50% of its lifetime, thus suggesting the need to avoid toxicants as best as possible by using the devices with high e-liquid levels in cartridges and tanks. Also, as shown in the supplementary file, usage of the device at 45 degrees inclination in general produces less carbonyl yields. The authors also stress the use of air blanks to avoid misrepresentation of the data in laboratory testing.

The study confirms facts that follow from the considerations we have presented in Section 2 on an optimal regime for aerosol generation and the realistic usage of devices, namely: negligible to low carbonyl yields occur in low-powered devices tested with an appropriate verification of absence of dry puffs, under CORESTA or CORESTA-like puffing protocols that are appropriate for the design of these devices. The authors do recognize that such puffing protocols are inadequate for testing sub-ohm open tank systems, which as we have stated, drastically reduce the power ranges of the optimal regime and do not provide (specially at high power settings) sufficient airflows and puff volumes that these devices require for an efficient aerosol generation to be used for DTL vaping.

**Reilly et al.** The authors [19] examine carbonyls and nicotine yields, as well as free radicals in aerosol emissions from four different flavors of Juul devices. The four flavors available at the time of the study (currently only tobacco and menthol are available) exhibited no difference in nicotine yields (164±41μg/puff), formaldehyde (0.20±0.10μg/puff), acetone (0.20±0.05μg/puff) and PG/VG ratio (PG/VG 30:70). To quantify free radicals the e-liquid in the cartridges were refilled with nicotine-free PG/VG ratios 30:70 or 60:40 with or without citral, leading to a concentration of 5.85 ± 1.20 pmol/puff ∼$10^{11}$ nmol/puff (5–6 orders of magnitude below cigarette smoke). Juul devices produce free radicals and carbonyls at substantially lower levels lower than those observed in other e-cig products.

### 6.3. Studies Published in 2020

**Son et al.** This study [21] evaluated the effects of device settings, vaping topographies and e-liquid compositions on the levels of carbonyl compounds. For the tested power settings devices with bottom coils generated 10–10,000 less formaldehyde than cigarette smoke. As the authors argue, aerosol emissions are affected by the patterns of of EC usage: puffing parameters (puff duration, frequency, volume), power settings, coils and e-liquids. As a guideline to determine their experimental settings from a wider variety of these patterns, the authors resort to the same parameters they have used in previous studies [40,95], based on observational data obtained from the same sample of 23 recruited vapers (we review [40] in Section 8).

All experiments were conducted with values from this observational data. For power and puff volume, the median (average, 95% percentile) were 6.4 (14.7, 31.3) W and 90 (35, 170) mL, puff duration 2.0 and 3.8 s (24 s interpuff interval). The EC was also the same is in [40,95]: refillable tank with replaceable Nichrome heating dual-bottom coils with 0.8 Ω, with two batteries Apollo Valiant battery (Apolo E-cigarette, Concord, CA, USA) and Sigelei-100W battery (Sigelei US, Pomona, CA, USA), range of power outputs 3–80 W, with wattage obtained by varying voltage for the 0.8 Ω coil. Collection and analysis: DNPH cartridges and an HPLC/UV system. To assure better control, e-liquids were prepared in situ in three compositions 100% VG, PG/VG 50:50 mixture and 100% PG, with 8 flavors freshly prepared by adding 10% of the corresponding flavoring agents.

As expected, aldehyde yields increased with power (6.4 W to 31.3 W), with larger rates for PG and PG:VG than for VG e-liquids, since PG boiling temperature is lower and byproduct formation initiates at lower temperatures as power increases. Formaldehyde yields increased for all e-liquids at increasing power (6.4–31.3 W), but (as expected) with a larger rate for PG and PG/VG 50:50 e-liquids than VG e-liquids. Acetaldehyde did not increase in 6.4–31.3 W in the VG e-liquid, but increased 2.7 and 8.5 times in PG/VG 50:50 and PG e-liquids. Acrolein yields increased 2 times between 6.4 W and 31.3 W. Fruit flavored e-liquids produced higher formaldehyde yields than mint/menthol and creamy/sweet flavored ones.

In terms of vaping topography, formaldehyde yields increased with increasing puff volume (35 mL to 90 mL) for fixed puff duration, but not significantly in 90–170 mL, increasing also with puff duration for fixed volume. However, as shown in their Table 3 for a pure VG e-liquid at 6.4 W, these combinations of puff volume and duration do not involve significant increase of aldehyde yields: for puff duration increasing 2 s to 3 s at average 90 mL puff volume formaldehyde goes from 790.0±32.3 to 903.0±56.2 ng/puff, with much smaller yields in all parameters for the remaining aldehydes.

This study is valuable because the authors have made an effort to incorporate in their experimental design a much wider set of vaping parameters (puffing, power settings, e-liquids, flavors) than most emission studies, which simply choose a fixed set for the whole testing. However, the authors’ choice of parameters is still too limited, even if referred to the data of the small sample of 23 vapers they are considering. For example, instead of considering e-liquids with pure PG and VG (which are not realistic) a better choice would have been PG/VG 30:70 and PG/VG 70:30 mixtures.

Also, Table S2 of their Supplementary File shows that up to a 75 percentile of the 23 vapers use power settings below 13 W, while the upper value 31.3 W corresponds to a 95 percentile (one of the 23 vapers). Also, the authors mention in [95] that “These selected power output levels (6.4, 14.7, 31.3 W) have been characterized as *safe*, *hot*, and *extremely hot* on a popular consumer Web site that provides users with vaping tutorials”. This is information from users based on their sensorial perception, it should not be dismissed (at least it should be considered). This information suggests that the lowest experimental value, 6.4 W, is representative (i.e., “safe”), not only of this sample but of consumers of this type of devices, while the upper experimental value 31.3 W is not only an outlier in this sample, but it is very likely a power setting that consumers would avoid (“extremely hot”).

Evidently, the possibility that 31.3 W could be an unrepresentative outlier (likely used in the reference sample by one or two vapers) should have been verified by inquiring with end users. Consultation with end users and considering the output from their sensorial experiences can be extremely useful to set up realistic and relevant experimental parameters (see [34,53]). This is important, since at 31.3 W the levels of all carbonyls (specially formaldehyde) that were found in this study are much higher (specially for the unrealistic pure PG e-liquid). Considering 31.3 W as still representative, but without verifying it, might lead to artificially high estimations of ranges of carbonyl yields and exposure levels.

Considering as the most realistic parameters the V:G 50:50 mixture at 6.4 W, the values of carbonyl yields reported in Table 4 of their supplementary file of experimental outcomes for the combinations of power and e-liquid composition, shows a maximal formaldehyde yield of about 1 μg/puf, which for 250 average daily puffs leads to a daily formaldehyde exposure of 250 μg/day which is close to the strict AFNOR and OEHAA thresholds (assuming 20 $m^{3}$ of daily breathed air), but well below the occupational PEL-NIOSH of 18.45 mg/day.

**Zelinkova and Wenzl** The authors [23] tested the Voopoo Drag with its 0.25 Ω and 0.5 Ω and Vaporesso SWAG device with 0.15 Ω and 0.5 Ω coils. The devices were filled with PG/VG 50:50 e-liquids and the puffing protocol was a CORESTA regime. Formaldehyde, acetaldehyde, acrolein, propionaldehyde, acetone, butyraldehyde, crotonaldehyde and 2-Butanone were quantified for each power tested and matched with the mass of e-liquid vaporized. Power levels were varied from the lower one recommended by the manufacturers to levels above their recommendation. Each experiment was conducted in triplicate. Although the usage of a CORESTA protocol for testing two sub-ohm devices is either unrealistic or only relevant for a minority niche of users (see Section 3 and Section 4), the study results are valuable, as they allow for the estimation of a maximal supplied power marking the outset of the exponential increase of aldehydes production. This study together with [22] illustrate the link between the optimal regime and a minimal aldehyde production (see Section 2).

**Talih et al.** The authors [22] examine and discuss the link between boiling processes and carbonyls formation. They tested 3 devices: a TF-N2 (0.12 Ω), a TFV12-Q4 (0.15 Ω) and a VF platinum (2.2 Ω), all filled with pure glycerol e-liquid, applying a 1 L/min airflow over 15 puffs of 4 s duration and 10 s interpuff interval (an excessively intense regime). This is an important study, which (as we discussed in Section 2) provides a significant contribution to the understanding of the physical processes of an overheating regime linked to film boiling. Although its experimental setup is unrealistic: two sub-ohm devices tested under a CORESTA-like regime with intense puffing, with all 3 devices tested at maximal power above the manufacturers recommendations, the authors’ results illustrate that maximal supplied powers leads to an exponential increase in carbonyl production, specially formaldehyde, whereas these byproducts remain at minimal levels under specific power ranges that define optimal operational conditions.

**Uchiyama et al.** The authors [24] examine the effects of power and temperature on the generation of byproducts resulting from the thermal degradation pathways of PG and VG from 3 comercial devices. The 3 tested devices were not fully identified (no information whatosoever is supplied on models and brands), but from the described characteristics of the mods and atomizers it is evident they are powerful sub-ohm devices. For their denomination as Brands A, B, C respectively, battery voltage (V): 3.7, 3.7, 37, power range (W): 1–75, 7–75, 7–85, coil type and resistance (Ω): stainless steel 0.1–3.5, stainless steel 0.1, stainless steel and zinc alloy 0.3–3, recommended power range (W): 40–50, 15–60, 30–60. New atomizers were used in all e-cigarettes. Only one commercially available e-liquid consisting of PG (approx. 30%), and VG (approx. 70%) containing nicotine (approx. 0.3%), menthol, and apple flavor was used for all e-cigarettes.

Aerosol generation and collection was conducted according to the CORESTA protocol: 55 mL puff volume, 3-s puff duration, and 60-s puff interval. The latter interval was modified from 30 s to 60 s, since puffing had to be interrupted after approximately 10 puffs because of overheating and so the EC switch was turned on 2 s before puffing and turned off 10 s later. Analysis was conducted with QP 2010 Plus GC/MS and LC-20(HPLC) systems (Shimadzu, Kyoto, Japan).

Generation of carbonyl byproducts was very low with total particulate matter (TPM) 13 mg/15 puffs at supplied power of 10 W, but above 40 W byproduct generation exponentially increased. Testing the devices at 50 W shows (their Table 4) that device B emits much higher mass levels (in μg) in the gas phase than those of tobacco smoke (CM6 cigarette) of formaldehyde (2300±220 vs. 15±0.5), acetaldehyde (1800±580 vs. 1200±150) and acrolein (830±87 vs. 120±3.2).

The authors recognize that such high outcomes are associated with high temperatures (determined by temperature programs of the mods but not validated by the authors) exceeding 500 °C at 60–75 W reached by the atomizer of the device B, while the maximum temperature of the atomizer Brands A and C was approximately 250 °C with small variations above 40 W (this differences in temperatures are displayed in their Figure 7), While formaldehyde, acetaldehyde and acrolein reached for Brands A and C much lower levels than those with Brand B, these levels are still comparable to those of tobacco smoke (in their Table 4).

Evidently, several flaws can be identified in this study. Failure to properly identify the devices makes it more difficult to interpret outcomes and prevents any replication of the experiments. An odd result is finding formaldehyde split in the gas and particle phases, when its high volatility suggests it should be predominantly in the gas phase (an explanation of this odd result in terms of the aerosol collection methods was suggested in [30]).

However, the main shortcoming of this study is the usage of a CORESTA puffing protocol for testing sub-ohm devices at high power settings, as this leads to user irrelevant experimental conditions that are prone to overheating, as the authors recognize when setting up the puffing procedure sequence by turning off the device puffing 10 s after each puff. While carbonyl outcomes from devices Brands A and B were much lower, they were also extremely likely artificial overestimations due to testing under inadequate parameters. The authors’ risk assessments are not relevant for real life vaping, as end users of these devices vape the DTL style with airflows and puffing volumes far larger than those of CORESTA protocol used to test them. A final remark, the authors refer to usage of EC’s as “smoking” and vaping aerosols as “smoke”. This a profoundly mistaken and misleading terminology.

**Cunningham et al.** This extensive study [25] by industry funded authors (British American Tobacco BAT) analyzes toxicant content in EC emissions from five EC manufactured by BAT, looking at the effects on the emissions from the variation of wicks, atomizer coils and benzoic acid content. After quantifying 97 aerosol constituents and 84 smoke compounds, 16 of the 19 HPHCs identified by FDA were absent in the emissions of all tested ECs. A comparison with two tobacco cigarettes showed that levels of the nine World Health Organization (WHO) TobReg priority cigarette smoke toxicants were more than 99% lower in the emissions of the ECs. No evidence was found of toxic byproducts formed from the thermal decomposition of benzoic acid in the e-cigarettes tested or from enhanced thermal decomposition of propylene glycol or glycerol by the nickel–iron coil.

The tested devices were BAT products: Vype ePen2 and Vype ePen3 (both Nicoventures Trading Ltd., Blackburn, UK). The study tested the Vype ePen2 at high power setting 4.4 W, but a low one 2.8 W is available. The device is button activated and is formed by a reusable section with a 650-mAh rechargeable battery and a disposable flavor cartridge, a silica rope wick and an NiCr coil. The Vype ePen3 operates at 5.9 W with a NiFe coil resistance 1.95–2.36 Ω, it is a closed system with a rechargeable 650-mAh battery and a flavored e-liquid pod of 2 mL capacity. The devices contains protect circuit board (PCB) to prevent over current and over charging. A fully charged battery provides 200 puffs.

The devices use e-liquids with different ratios of nicotine vs. benzoic acid and slightly different PG/VG ratio. For the comparison of the aerosol chemistry the ePen2 and ePen3 are filled with Blended Tobacco (18 mg/mL nicotine) and only the ePen3 with Master Blend (18 mg/mL nicotine with medium benzoic acid). The two cigarette products were: Kentucky reference 1R6F and Benson & Hedges Skyblue (Japan Tobacco International).

EC devices were tested with the puffing protocols of the CORESTA method 81 EC. For the tobacco cigarettes Canadian Modified conditions (55-mL puff volume, 2-s puff duration, 30-s interval, vents blocked). Aerosol collection: glass fiber filter disc (pad) followed by an impinger. Analysis by GC-MS. The authors provide measurements of background air/method samples and emphasize their importance to avoid confounding the quantification of toxicants at low concentrations with contamination from laboratory air and analytical methodology equipment and reagents.

Quantified outcomes are displayed as mass per puff for the ECs and tobacco cigarettes (Tables 3 and 4). For the ECs, besides aerosol collected matter, water, nicotine and the solvents PG and VG, most carbonyls appear BLD or NQ, with formaldehyde, acetaldehyde, acetone, methylglyoxal, isobutyraldehyde appearing at ng/puff levels. The remaining compounds (triacetin, menthol, PAHs, VOCs, TSNA, phenols, flavorants, acids and CO) are also BLD or NQ. The authors display the outcomes of same compounds for EC emissions as mass normalized by nicotine in their Tables 4 and 5. Depending on the nicotine concentration in the e-liquids, the ePen2 had 3–7 times lower nicotine yields per-puff than the ePen3 and 81% lower than those from both cigarettes, while the ePen3 with high levels of benzoic acid produced larger nicotine yields than the cigarettes.

The authors discuss various forms of comparing EC emissions with tobacco smoke, all of which showing a 2–3 orders of magnitude reduction in toxicant content. The lack of detection of benzene, phenols and PAHs rules out degradation products from benzoic acid, while absence of byproducts of wick materials (cotton and silica) in both EC devices disproves the hypothesis that silica is thermally more stable than cotton and provides evidence that wicks of both devices are stable under standard EC operating conditions.

**Noël et al.** The authors [26] examine the production of three aldehydes (formaldehyde, acetaldehyde and acrolein) from an unspecified EC device, undertaking a comprehensive cytotoxicity analysis on gene expression in human bronchial epithelial cells exposed at the air–liquid interface to the device emissions.

The authors do not disclose the device model and brand, they only mention that it operates with atomizers set up with 9 distinct resistance/voltage combinations: 0.15, 0.5 and 1.5 Ω and 2.8, 3.8 and 4.8 V, which leads from Ohm’s law to these power ranges for each voltage: 52, 96, 153 W for 0.15 Ω, 16, 28, 46 W for 0.5 Ω and 5, 10, 15 W for 1.5 Ω. E-liquids with 36 mg/mL of nicotine were used (to mimic exposure of heavy smokers), PG/VG 50:50 ratio, and with either butter or cinnamon flavors. The puffing protocol was CORESTA 81: 3-s puff duration, and a 55-mL puff volume every 30-s. Samples were collected in 10-puff sessions. Quantification by gas chromatography with a flame ionization detector (GC- FID). Analysis HPLC.

Nicotine, acetaldehyde, formaldehyde, and acrolein levels (μg/puff) were flavor dependent. Large increase occurs when comparing 0.15 vs. 1.5 Ω at 4.8 V (15, 46, 153 W). Lesser increase was found for cinnamon flavor (acrolein was below LOD). Carbonyl yields were low for 1.5 Ω at all voltages (5, 10, 15 W). From the bars in their Figure 1, the three aldehydes have negligible levels (approx. 0.5 μg/puff) for all tests with 1.5 Ω and all voltages, with formaldehyde, acetaldehyde and acrolein, respectively, reaching 18, 10, 10 μg/puff for the combination 0.15 Ω at 4.8 V (153 W), with values for 0.5 Ω closer to those with 1.5 Ω.

The authors claim that sub-ohm vaping induces flavor dependent detrimental effects to human lung epithelial cells. They conclude that taking together their experimental results could help policymakers to “take the necessary steps to prevent the use or manufacturing of sub-ohm (i.e., 0.15 Ω) atomizers”, as their emissions induce flavor-specific detrimental effects on lung cells due to cytotoxicity, enhanced oxidative stress, low levels of nitric oxide, diminished transepithelial electrical resistance (TEER), and altered expression of key genes associated with biotransformation, oxidative stress, and inflammation, all this besides cellular toxicity via oxidative stress mechanisms. Further, they claim that their data also suggests that ECs may not be a “safe” alternative to conventional cigarettes.

Evidently, the authors are issuing completely unwarranted and disproportionate statements. The claim that the type of laboratory cytotoxic experiments they have performed can somehow predict actual clinically verified harm in sub-ohm vapers, which would merit such a harsh regulation, is extremely speculative. The resulting increase of aldehyde levels, as well as their connection with deleterious cytotoxic effects from the sub-ohm device, simply follow from the authors’ inappropriate and unrealistic experimental design: using a CORESTA puffing protocol to test this type of device at high power settings (153 W), which as we explained in Section 2 are the testing conditions that lead to overheating and possibly dry puff, producing an aerosol that end users most likely would find repellent.

**Mallock et al.** This German study [27] compared the US and European versions of Juul. While the early European version did not compensate for a much lower e-liquid nicotine concentration in this version, the modified version shows an increased vaporization that provides a better approximation to the nicotine delivery of the US version. Notably, carbonyl levels remain comparable to those of the US product. In general, carbonyl and other emissions byproducts are detected in Juul devices in lower levels than in other pod devices.

### 6.4. Studies Published in 2021

**Chen et al.** This comprehensive study [28] of Juul emissions by from Juul Labs, tested 4 Juul devices in terms of 4 product combinations available in the US market in 2021: nicotine concentrations 35/59 mg/mL in two favors: Virginia Tobacco (VT) and Menthol (Me). Carbonyls are in the Group I of analytes based on FDA guidance for in its Pre Market Tobacco Authorization (PMTA) process for vaping products. Aerosol was collected for Group I only in the “nonintense” (NI) regime with 3 s puff duration and 30 s interpuff interval. Ten replicate measurements were performed from each of each product combination. More than 40 of the 53 targeted analytes were below detection or below quantification. Mass per puff was analyzed over three 50-puff collection blocks: beginning, middle and one at the end. The outcomes were displayed in Table 3, showing the largest mass yields for formaldehyde and acetaldehyde at ∼$10^{-2}\;\mu$g for all product combinations.

**Crosswhite at al.** This study [29] was also funded by Juul Labs. It applied a nontargeted analysis to obtain a more complete list of aerosol constituents in the aerosol generated by the 4 varieties of Juul devices: Virginia Tobacco pods with 3.0% and 5.0% nicotine concentrations (VT3 and VT5). Aerosol was generated in sequential 50 puff blocks for a nonintense (3 s puff duration, 30 s interpuff lapse, 55 mL puff volume) and intense (6 s puff duration, 30 s interpuff lapse, 110 mL puff volume) regimes.

The analysis employs two complimentary nontargeted analytic methods: GC–MS methods optimized and adequate for analysis of for volatile/polar, including flavor and aroma compounds, together with LC–MS-based methods amenable to characterize semi/non/volatile, semi/non/polar and higher-molecular-weight compounds that might be contained in the liquid droplets making the particulate phase of EC aerosol. hieve a quantity estimation across multiple compounds. While these complementary analysis methods cover a broad chemical space, they cannot detect all chemicals in the aerosol (metals and nonionizable compounds). Aerosol trapping methods were adapted for each analytic technique. Blank samples were also analyzed.

Nicotine, PG, VG and benzoic acid were not detected. All detected compounds were above 0.7 μg/g for GC–MS analysis and above 0.5 μg/g for LC–HRMS analysis and differing from blank measurements were identified and semiquantified. Tentatively identified analytes were grouped in five groups: flavorants, HPHCs (from the FDA tobacco product supplied list), extractable and leachables, byproducts of chemical reactions and unidentifiable compounds. For VT3 the five groups formed 0.23% (intense) and 0.2% (non intense) of the aerosol mass, itemized as flavorants: 70% (intense) and 75% (nonintense), reaction byproducts: 16% (intense and nonintense). HPHCs were not detected in the nonintense regime. Similar outcomes were obtained for the VT5 in both intensity regimes. The numbers of detected compounds were 88 (VT2) and 91 (VT5), of which 67 are common in both intensity regimes, while compounds common with the 5162 compounds of tobacco smoke were 29 (VT3) and 32 (VT5).

**Li et al.** The authors [30] tested mainstream aerosols from a third generation device, Evolv DNA 75 color modular vaping device (Evolv LLC., Hudson, Ohio) with replacement single mesh vaping coils (SS316L, FreeMax Technology Inc., Shenzhen, China) that have a coil resistance of 0.12 Ω.

Emissions were tested for puffing parameters close to the CORESTA protocol: fixed flow rate 1.186±0.002 and puff volume 59.3±0.1 mL, but with variable puffing rate (2, 3, 4 puffs/min) and puff duration (2, 3, 4 s) at a fixed flow rate, both at 191 °C, 3 s puff and e-liquid with PG/VG = 30/70 and 3 mg/mL nicotine. Temperature was set for the tests at 157, 191, 216, 246 and 266 °C with the temperature control software supplied by the manufacturers. The authors recognize that this modified CORESTA protocol cannot be extrapolated to real vaping scenarios, but claim that increasing puff duration and controlling temperature can somehow compensate this limitation. However, it is unrealistic to keep fixed a CORESTA airflow and the temperature (assuming the device temperature control is accurate) while increasing puff duration. As we elaborate in Section 2 and Section 12, this is the main drawback of this study.

The chemical characterization of carbonyl byproducts is undertaken by targeted and nontargeted analyses using LCHR-MS (liquid chromatography high-resolution mass spectrometry), GC gas chromatography, besides in situ chemical ionization mass spectrometry, and gravimetry. The authors provide a comprehensive discussion of the thermal degradation reaction pathways involved in carbonyl production in the normal temperature range of realistic vaping (below 266 °C), arguing that the heat-induced dehydration mechanism is dominant over the path of H-abstraction by radicals such as OH, the latter playing a minor but not negligible role.

The results of the study reinforce known outcomes: most aerosol components are volatile or semivolatile, with over 99.5% of emissions made of PG and VG in both aerosol phases. The study finds that PG mostly tends to be found in the gas phase, with the particle phase containing substantial part of VG and all nicotine (the nicotine phase partition depends on the e-liquid PH). Volatile carbonyls (including formaldehyde) tend to be in the gas phase. Other outcomes are:

- The temperature dependence of carbonyl production is very sensitive to the coil metal alloy (the authors’ Figure 2), an expected result given the different heat conducting coefficients of these metals.
- The mass yields (in μg/puff) displayed in their Figure 3 show formaldehyde, hydroxyacetone, acetaldehyde, acrolein, and propionaldehyde, characterized by an exponential dependence on temperature that is seen to become steep at the upper temperature range (266 °C), while the dependence is linear for acetone, dihydroxyacetone, and glyceraldehyde.
- The PG/VG and nicotine ratios and nicotine proportion in the particle phase closely mirrors the PG/VG e-liquid ratio, with the 30/70 mixture (at 191 °C) leading to roughly 3/4 VG and 1/4 PG with 0.3% nicotine.
- As expected the mass concentration of 7 carbonyls increase with an increasing puff duration at same temperature (191 °C) and with fixed airflow (which makes these values unrealistic).

These results are consistent with the reaction modeling suggesting a higher efficiency of the heat-induced path at the tested temperature range, while the linear dependence (which may become exponential at temperatures higher than those tested) is consistent with the radical pathway dominating at temperatures above 360 °C. Although computerized modeling supports the heat-induced pathway from VG to be more efficient than the radical pathway at normal EC usage temperatures, formaldehyde, hydroxyacetone and acrolein can be formed from either pathway and from both PG and VG.

In discussing health impacts, the authors recognize that the doses for both solvents PG and VG are below toxicological makers and that the carbonyl concentrations they found are way below those of tobacco cigarettes (even in their higher range of tested temperatures). However, they develop questionable speculations on inhaling excessive doses of acrolein from the fact that this aldehyde is mainly a byproduct of VG, which becomes the dominant compound in the emissions as vaping proceeds.

Assuming an 8:1 ratio of VG to PG mass aerosolization for a PG/VG 30:70 mixture, they estimate (based on the outcomes they found in the study) that only 30–40% of the e-liquid will be consumed (well before liquid depletion) when it becomes entirely VG with likely a high acrolein content. This is very questionable, first because this hypothetical acroleine exposure is not applicable to actual vapers, since (as the authors admit) the puffing setup that they used cannot be extrapolated to real vaping scenarios (we discuss this issue in Section 12). Second, since sub-ohm tank devices are normally vaped with PG/VG 30:70 ratios and low nicotine concentrations for direct to lung style, serious deleterious effects from such an acrolein excess would have likely been noticed by end users who (following the authors’ hypothesis) would end up vaping almost pure VG at high power ranges. However, so far the main reported concerning carbonyls are formaldehyde and acetaldehyde, with acrolein playing a minor role and (as far as we are aware) there is no evidence that sub-ohm vaping of VG dominated e-liquids intrinsically produces more adverse respiratory effects than other vaping styles and e-liquids.

**Yan et al.** The authors [31] apply orbitrap MS for a nontargeted analysis of EC emissions. They identify more than 30 “features” characterized by pairs of the mass to-charge ratio of the compound and the retention time. Compounds are identified containing nicotine and PG (NIC-PG) with increasing abundance relative to nicotine increasing supplied power.

Devices: OD1: an iStick 25 (Eleaf, Shenzhen, China) with power range 1–85 W and equipped with a HW2 coil (recommended range 20–70 W), and OD2: a SMOK Alien 220 Mod device (Shenzhen IVPS Technology Co, Shenzhen, China) with P = 6–220 W and equipped with a TFV8 Baby Tank and a SMOK V8 Baby-Q2 coil (recommended power range: 20–50 W). E-liquid was VG/PG 80:20 mixture. Aerosol was collected by a system of tubes and pipettes to a peristaltic pump. The devices were tested at high powers, OD1: 20, 40 and 80 W, OD2: 40, 120 and 200 W. The puffing protocol was: 4 s puffs, interpuff time 26 s at a flow rate of 1 L/min.

Except for the use of a different analytic technique (orbitrap MS) and a nontargeted approach, the authors used exactly the same experimental setup (devices, power settings, puffing parameters) as a study that we reviewed in [5] by Zhao et al. [96], which tested by means of the same low airflow CORESTA-like protocol the same high-powered sub-ohm devices at high power settings, even well above the settings recommended by the manufacturers. As we argue in Section 2, this experimental setting is a blue print for detecting large byproduct yields produced by laboratory testing under unrealistic and clearly overheating conditions that are prone to produce large levels of toxic byproducts. As with the sub-ohm devices tested in the study reviewed in [5], the experimental results of this study have no relevance for end users.

**Tehrani et al.** The authors [32] apply LC-HRMS, a sensitive analysis technique, to a nontargeted study of e-liquids and EC aerosols from 4 devices. The number of detected compounds and the proportion of combustion associated hydrocarbons in e-liquids increased when in aerosol form. Lipids and hazardous additives and contaminants, such as tributylphosphine oxide and the stimulant caffeine were also detected in e-liquids and aerosols.

The authors tested the following 4 devices: a third-generation modifiable-power (Smok ProColor 225W with TFV8 Big Baby Beast Tank, Shenzhen Ivps Co., Ltd., Shenzhen, China), two fourth-generation cartridge (“pods”): Juul, Juul Labs, San Francisco, CA and Vuse Alto, British American Tobacco, London, UK), and a disposable pod device (Blu Disposable, Imperial Brands, Bristol, UK). E-liquids covered a wide range of nicotine levels (whether base or salts) but only with tobacco flavor.

Aerosol was generated at 1.1 L/min airflow with puff topography based on the International Organization for Standardization 20768:2018 method 1541: 3 s puff duration and a shortened interpuff interval of 10 s. The shorter interpuff interval was found to be necessary to produce sufficient condensed aerosol sample volumes for Blu, Juul, and the PG/VG base e-liquid. Three blocks of consecutive 100 puffs each were machine puffed for each device, recharging after each block, with aerosols generated from a single pool of e-liquid for each product for the 3 blocks. Slightly higher aerosol mass was generated in the final 100-puff interval for the Juul, whose maximal set by manufacturers is 200 puffs.

It is not surprising that such a vast array of compounds were detected, given the high resolution of LC-HRMS and the enormous amount of generated aerosol from such an extremely intense puffing regime: 3 blocks of 100 puffs taken each 10 s. Since demographic studies of vapers in natural conditions [72,73,74,75] show on average 200–300 daily puffs, such a regime would involve compacting all daily puffing during 12–16 h of wakefulness into 30–50 min. This is evidently an unrealistic experimental setup geared to detect as much compounds as possible, even if this is achieved under conditions completely unrelated and detached from real life.

Besides being unrealistic, this regime is an artificial way to magnify detection of byproducts in emissions, even from the trapping material or the environment. As shown in the study by Belushkin et al. [18], production of byproducts in the aerosol significantly increases as a device is consuming e-liquid that is progressively “aging”, even without depletion. This aging can be critical for low-powered pod devices being puffed with the same e-liquid 300 times every 10 s.

Regarding the Smok ProColor 225W with a TFV8 Big Baby Beast Tank, a powerful sub-ohm device, the authors fail to disclose the power settings and coil resistance with which it was tested, an essential information to interpret and possibly replicate their results. Also, the use of an airflow of 1.1 L/min is completely inappropriate for such a device designed and used for DTL vaping. Testing this device with this puffing regime most likely leads to overheating conditions even if puffed within the recommended power settings (see Section 2). Furfural and various fatty acids are byproducts of cellulose (wick) pyrolysis (see Section 3), but are also among the compounds detected in this study, which would confirm testing under dry puff conditions.

The authors present a very detailed examination and classification of the detected compounds, validation tests and calibrations. However, this comprehensive study is completely unrealistic and irrelevant for end users.

**Cancelada et al.** The authors [20] quantified HPHCs in aerosols emissions generated using a SMOK V8 kit designed with a TFV8 Big Baby tank and five coils M2 (0.15 Ω and 0.25 Ω), X4 (0.15 Ω), T8 (0.15 Ω) and Q2 (0.6 Ω) filled with a commercial e-liquid Euro Gold from Naked 100. The experiments were conducted using a fixed nominal voltage of 3.8V (i.e., 98 W for 0.15 Ω coils, 58 W for 0.25 Ω coil and 24 W for 0.6 Ω coil) varying puff volume (50 mL, 100 mL, 250 mL, 350 mL and 500 mL) and a fraction of airflow vent system opening (0%, 25%, 75% and 100%). The first series of experiments reported the masses of e-liquid vaporized (MEV) according to the puff volumes for the different opening fractions tested using the M2 0.15 Ω. Because the results are close to each other (except at very low puffs volume), the authors reported that the puff volume and opening fraction did not affect the mass of e-liquid vaporized. As we argue in detail in Section 2, this interpretation of the results is erroneous, as the experiments were conducted with a single power. This is even more confusing, as the M2-0.15 Ω coil is normally recommended by the manufacturer to operate between 25 W and 80 W and the one tested here was running at 96 W (based on manufacturer information).

The authors further graphed an open airflow system vs. puff volume in a grid, with formaldehyde levels quantified and reported in terms of the mass of e-liquid vaporised in the emissions. The resulting graph shows some higher formaldehyde levels with small puff volume and reduced airflow than with high puff volume and fully open vent system. Even more interesting, iso-lines can be estimated with a picture at 0% and 50 mL and tends to a very low limit approaching 500 mL and/or 100%. The authors argue that increasing airflow rate can reduce the degree of decomposition of e-liquid components. In reality, changing the puff volume or the airflow opening fraction modifies the same parameter: the speed of air, as they change the pressure drop in the device. With the characterizations of the air resistance for each opening fraction these results would have highlighted that the iso-values are due to experiments carried out with the same speed of air and the same heat exchange by forced convection. The remaining graph would be a classic one with the speed of air in the x-axis and the formalehyde ratio in the y-axis. As for MEV, the interpretation of the results is confusing because it was obtained with a single fixed power.

Finally, the authors tested the five coils using two opening fractions 50% and 25% with a 50 mL puff volume and reported the MEV and the quantities of some HCHPs with 50% fraction. Based on the previous experiments, the authors would provide results obtained with a CORESTA-like vaping regime unless they made experiments using a puff volume (i.e., airflow rate) consistent with DTL. The MEV of the 0.6 Ω coil (24 W) is lower than 0.25 Ω (58 W), itself lower than 0.15 Ω dual and quadruple coils (96 W), still lower than the octuple coils. As these classification is now surprising, it should be noted that they are initially recommended for power range of respectively 40 W–80 W (Q2), 30–50 W (M2), 25–80 W (M2), 30–70 W (X4) and 50–110 W (M2) highlighting that Q2 was tested below the requirements, T8 under the range required and M2, X4 above the requirements. Based on this observation, it is also not surprising that so high levels of carbonyls were found for M2 0.25 Ω and that 0.15 Ω dual coil has high deviations compared to the results of the others coils suggesting that dry puff occurs for one device.

### 6.5. Studies Published in 2022

**Xu et al.** The authors [33] quantified HPHCs in aerosols emissions of four market-leading flavoured e-cigarettes available in the Chinese market. Levels of eight carbonyls, five volatile organic compounds (VOCs), four tobacco-specific nitrosamines (TSNAs), 16 polycyclic aromatic hydrocarbons (PAHs), and seven heavy metals where quantified and compared with their presence in mainstream tobacco smoke. Small variations of mass yields of carbonyls were found among the different ECs, but the vast majority of targeted HPHCs were either undetected or found in significantly lower yields than in cigarette smoke (commercial or reference 3R4F).

Two devices were tested, each with nonrefillable cartridges in two flavors: RELX Classic (mung bean and tobacco) and RELX Infinity (coke and watermelon), both operating at 6.5 W. Aerosols were generated and collected by 100 puffs with the CORESTA Recommended Method No. 81: a square-wave puff profile, 55 mL puff volume, 3 s puff duration, and 30 s interpuff interval. Aerosol condensate was passed either through a collection vessel containing suitable solvent for the analysis of carbonyls, VOCs or heavy metals (lead, stibium, arsenic, nickel, chromium, cadmium, and mercury). TSNAs and PAHs were collected through a Cambridge filter pad. For comparison with tobacco smoke a cigarette was counted as 10 EC puffs.

Quantification of formaldehyde, acetaldehyde, acrolein, acetone, propionaldehyde, butanol, butyraldehyde, and butanone was based on CORESTA Recommended Method No. 7414 and the AFNOR XP D90-300-3 standard. Samples were analyzed by ultraperformance liquid chromatography (UPLC). The study also targeted and quantified VOCs, TSNAs, PAHs and heavy metals. The mass yields as μg/100 puffs and μg/10 cigarettes were displayed in Table 2.

For carbonyls, the largest mass yield (ng/puff) in the ECs was for formaldehyde: 0.017–0.15, acetaldehyde: 0.017–0.092, acetone: 0.005–0.048, acrolein: 0.015–0.042, with the remaining carbonyls below detection. VOCs, TSNAs, PAHs were all below detection limits, while negligible yields were quantified for heavy metals (the largest for nickel at 1.78 ng/puff). Carbonyls yields were three orders of magnitude smaller than their corresponding yields in tobacco smoke.

In the neutral red uptake and Ames assays, aqueous extracts of the e-cigarette aerosols did not induce obvious cytotoxicity or mutagenicity, whereas CS aqueous extract showed dose-related cytotoxicity and mutagenicity.

**Visser et al.** The authors [34] present a ‘human volunteer-validated’ approach that can provide user relevant conditions in laboratory testing of EC emissions. The study matches the dry puff assessment from 13 volunteering experienced vapers with carbonyl detection laboratory testing. This approach can reduce the possibility of reporting excessively high toxicant levels that emerge in tests conducted under unrealistic conditions, producing EC aerosols that end users would find unpleasant and even repellent (specially the dry puff ’burned out’ sensation).

Vapers used the same EC devices (JustFog Q16C with 1.2 and 1.6 Ω coils and eLeaf Pico batteries). Power ranges recommended by the manufacturer were 6.4–12.1 W (1.6 Ω coil) and 8.5–16.1 W (1.2 Ω coil). The e-liquids were: menthol, vanilla and fruit, with PG/VG ratio 50/50, 30/70, 30/70 and nicotine levels 0, 0, 3 mg/mL, respectively. The vapers were instructed to vape in ‘mouth to lung’ style (to avoid as much as possible excessive deviation in airflows and puff volumes), sampling six combinations of coils, e-liquids and power levels (10 W–25 W in 3 W steps), reporting the absence/presence of a dry puff sensation through a 100-unit visual analog scale. Every combination was classified as either “dry puff flavor” (10% of the puffs reporting this sensation) or “no dry puff flavor” (otherwise).

As expected, dry puff sensation was not reported in any coil/liquid combination at lower power ranges 10–13 W, but above 13 W a complicated pattern emerged that depends on the e-liquids and coils. In parallel with this assessment, laboratory testing was conducted for the same combinations of coils, e-liquids and power settings assessed by the vapers, targeting carbonyl compounds through a HPLC-DAD (high-performance liquid chromatography—diode array detection) analysis. The puffing parameters were blocks of three 3-second puffs with 1-minute intervals and puff volume of 55 mL. The analysis detected only at higher power ranges 11 carbonyl species: formaldehyde, two acetaldehyde isomers, acrolein and lactaldehyde (the remaining were not identified). These compounds were denoted “dry puff markers” and a cutoff for each one was defined as its maximal quantified value (in μg/puff) in the power range (in each combination of coils/liquids) where the vapers assessed “no dry puff flavor”. For every coil/liquid combination, outcomes of these 11 markers above the cutoff were regarded as dry puffs (a very conservative approach, since the vapers did not report a dry puffs in 90% of the puffs).

The criterion to test the matching between vapers’ sensorial assessment and the chemical analysis was given by 11 carbonyl markers being above their cutoff values, which identified the corresponding coil/liquid/power combinations as dry puffs. This matching was consistent for 83% of puffs with 17% false negatives, with the largest divergence occurring in the fruit flavored e-liquid with the 1.6 Ω coil (60% dry puffs from the carbonyl test vs. 20% for the sensorial evaluation at about 20 W).

This study represents a fist step in the incorporation of end user input in the improvement of laboratory testing of EC emissions. The authors recognize the obvious limitations of the study (small sample of users, e-liquids, coils and a single EC device, subjectivity of sensorial validation). However, the study has other limitations not mentioned by the authors. The dry puff is not a sudden isolated event, it can emerge gradually, with overheating before a dry puff producing variation of sensorial experiences in users (the authors did not verify e-liquids depletion, so it can not be ruled out that users reported these variations). Evidently, as the authors suggest, studies with larger samples of vapers and devices should follow since human assistance can increase the accuracy and quality of emission testing.

## 7. Carbon Monoxide (CO)

The present section provides a review of three studies that detected CO in EC aerosol emissions. Since CO is a hazardous byproduct of incomplete combustion, it is important to understand under which experimental conditions it has been detected when testing ECs.

**Casebolt et al. (2019)**. The authors [35] used a Cleito device with a 0.2 Ω coil filled with two commercial e-liquids of 50:50 PG/VG ratio. Despite the fact that this device is designed for DTL inhalation, the experimental airflow rate was 0.85 L/min, which is inconsistent with real use of the device (it is even below that of the CORESTA protocol for the low intensity vaping regime ISO20768: 1.1 LT/min). The recommended power specifications for this device can be found in the internet: between 55 and 70 Watts. Ignoring this information they conducted the experiments applying powers from 40 to 180 Watts by steps of 20 Watts. Their results reveal an exponential increase in the maximal concentration of CO from 60 Watts onwards, though it is close to zero for power below 60 Watts. Casebolt et al. [35] concluded by an alarmist warning on CO production in commercial devices, despite the fact that their results show that CO was not quantifiable under the normal operational power of the devices, only under unrealistic tested conditions.

**El-Hellani et al. (2019)**. The authors [36] used a rebuildable atomizer to test the presence of CO in emission from sub-ohm electronic cigarettes. For this purpose they designed a coil from different alloys (nichrome, nickel, stainless steel and Kanthal) by wrapping a 24 or 26 Ga wire (diameters of 0.511 mm and 0.405 mm) around an inner diameter of 3 mm leading to 10 or 13 wraps. Japanese cotton was wetted with a 30/70 PG/VG e-liquid ratio and an air flow rate of 1 L/min was applied. All their coils had the same surface, though their geometric shape was different. In their Figure 3 they reported the detection of CO in the aerosol when applying powers from 25 to 175 Watts by steps of 25 Watts. No CO was detected below 100 Watts and levels started to be detected from 125 Watts with an increasing peak according to the supplied power. Then, in their Figure 1, they compared the use of different coil geometries (keeping their surface constant) at 125 Watts. For the same geometry (denoted by A in their paper), CO was quantified with its reported values displaying a high dispersion, thus suggesting that this is not a reproducible experiment. This might be due to the fact that 125 Watts is the minimal starting point of production. Finally, they designed an additional geometry to compare CO daily exposure at 75, 125 and 200 Watts to the CO exposure from a conventional cigarette. The geometry they used represents half of the initial geometry (5 wraps instead of 10), leading to a surface divide by 2. As boiling is a thermal phenomenon and the coil surface is a key parameter, the functioning limits using 5 wraps can be estimated by dividing them at 10 wraps by the same order of magnitude. Therefore, from the detection of CO using 10 wraps beginning at 100–125 Watts, we can assume that these limits would be between 50 and 63 Watts using 5 wraps.

The authors assumed glycerol pyrolysis as the source of CO production. Their results from an experimented study [97] on the degradation of glycerol in the gas phase shows production of CO and also CO${}_{2}$ from 675 C onwards, which are temperatures well above those of a vaping device in which glycerol is heated and vaporized from its liquid phase. As it reaches gas phase conditions, it mixes with fresh air and cools almost instantaneously. Rather, CO production can be explained by another source of pyrolysis. As film boiling is initiated, it creates a layer of gas around a small part of the wire creating an associated volume of dried fibers under conditions that are adequate for cellulose pyrolysis for which CO and CO${}_{2}$ formation is well documented and reported at temperatures above 400 C [98,99]. Therefore, there is a high probability that the authors detected CO under dry puff conditions that are repellent to end users.

**Son et al. (2019)**. The authors [37] investigated CO productions using a pod (JUUL), a ciga-like (V2 VMR products), a top-coil (Ego CE4) and a mod (Cleito 0.4 Ω). Ciga-likes are now obsolete and the Ego CE4 is no longer available due to capillary issues, but the Juul and Cleito devices are still marketed. The Cleito is recommended for DTL vaping and for power between 40 and 60 Watts. The airflow rate during the experiments (1.5 L/min) is inconsistent with the real use of the device. Together, the JUUL and Cleito produced a quantity of CO below 10 μg per puff, leading to a daily inhalation of CO below 2.5 mg (assuming 250 puffs per day). The WHO recommends a maximal allowed concentration of 103 mg/$m^{3}$ during 15 consecutive minutes (in a volume inhaled of 0.2 $m^{3}$ in 15 min). Even if a user vapes 250 daily puffs in 15 min (an impossible feat), the exposure concentration is 12 mg/$m^{3}$, representing 12% of the WHO recommendation. It is evident that spacing the puffs through a day leads to the dilution of local peaks of concentrations in CO and to an exposure concentration representing 2% of the daily requirement (7 mg/$m^{3}$). Using the most worrying result obtained in the paper, CO does not exceed this toxicological limit. In comparison with a conventional cigarette (10 cigarettes per day, 13 puffs per cigarette, 0.75–1.73 mg of CO per puff), a daily use of a vaping device leads to the inhalation of at most 1/161 of the CO inhaled from a conventional cigarette.

## 8. Reactive Oxygen Species (ROS)

Reactive Oxygen Species (ROS), specially Hydroxyls and OH compounds, in EC aerosol have been the subject of several studies [38,39,40,41,100,101] (we exclude [101], as its experimental procedure is too obscure and EC devices were not identified). Free radicals are an important group of potentially toxic byproducts, as they are a major constituent of reactive oxygen species (ROS) and reactive nitrogen species (RNS) responsible for oxidative stress affecting many physiological functions. We review five studies focused on free radical detection in EC aerosol emissions.

**Bitzer et al. (2018a)**. The authors [38] tested a powerful sub-ohm device: Wismec Reuleaux RX200S Mod, powered by three 18650 batteries and endowed with “constant control temperature” and “constant control power” modes, which allow the user to operate the device by fixing a target constant temperature or power in the ranges 100–315 °C and 1–200 W. The tests were conducted in both modes. As in previous studies [39,100], the authors conducted experiments with CORESTA-like puffing parameters: 40 puffs, 5 s puff duration, 30 s interpuff lapse and airflow rate of 500 mL/min = 8.33 mL/s.

The study spin trapped and analyzed free radicals through a nontargeted species approach by electron paramagnetic resonance (EPR). The detected free radical abundance was ∼$10^{13}$–$10^{14}$ molecules/puff. A previous 2016 study by the same group of authors [100] using a low-powered device detected an abundance of ∼$10^{13}$. These free radical levels are between 1/100 and 1/1000 of the free radical abundance of ∼$10^{16}$–$10^{17}$ molecules/puff detected in tobacco smoke with the same EPR technique (see [38,39,100]). Free radical production increased as follows:

- close to 2-fold between 100 and 300 °C under constant-temperature regime.
- at even steeper rate from 10 to 50 W under constant wattage, with coil temperatures higher than those of the constant-temperature regime.
- close to 3-fold in e-liquid mixtures with higher PG content in comparison with ratios of PG/VG 0:100 and PG/VG 100:0. This was associated with an increases in aerosol-induced oxidation of biologically relevant lipids.

These results show that reactive free radical production in e-cigarette aerosols is highly solvent. While radical production depended on aerosol generated at higher temperatures, disproportionately high levels were observed at close to 100 °C despite limited aerosol production.

However, there are various problems with the experimental setup. Coil temperature controls from instruments in sub-ohm devices are not very reliable: they are based on applying Fourier law to flat homogenous medium whose conductive properties are given by simplified empiric relations between power and resistance for different alloys (the formula in equation 1 of the study). To address this problem the authors should have provided an experimental validation of temperature measurements. There is also a conceptual problem with fixing the temperature (See Section 12), which is a thermodynamical intensive state variable whose evolution is determined (see Section 2) by the heat flux exchange balance between e-liquid vaporization close to thermal equilibrium and its condensation (and aerosol formation) by forced convection. Fixing the temperature at an arbitrary value (assuming it is experimentally validated and accurate) would force an isothermal process that is most likely, either incompatible with the heat exchange balance, or conducing to contrived conditions on coil resistance or power.

The constant power regime is more consistent, as power is an extensive state variable that can be set up externally. However, the authors used a CORESTA-like puffing protocols with an extremely small airflow rate (0.5 L/min). As we argue in Section 3 and Section 4, such diminished airflow is problematic for testing a powerful sub-ohm device, as it should lead to a severely narrowed power range of the optimal regime (see Figure 2), with a high likelihood that its upper power end (which determines the outset of overheating) lies within the 10–50 W ranges in which the authors conducted the tests. Not surprisingly, the authors detected a steep increase of free radical production as the device was tested along 10–50 W. The authors mention that this increase was due to high temperatures, but we would add that this is a further sign of testing in clear overheating conditions.

While the experimental settings of this study might allow for certain combinations of power, temperature and resistance that correspond to real life usage or at least conditions relevant to a majority of users, the authors do recognize (and we argue in Section 3 and Section 4) that it might also lead to combinations that are completely unrealistic or only applicable to a minority of vapers who might use sub-ohm devices for MTL style. This is a valuable study, but its experimental outcomes are likely applicable only to a minority niche of vapers (see Section 2, Section 3.1 and Section 12.1).

**Bitzer et al. (2018b)**. This is a study [39] by the same group of authors as [38], focusing on the effect of flavorings compounds on the production of free radicals. The authors use the same device as [38], a device endowed with a constant temperature mode that permits setting up its operation at a fixed temperature. The puffing protocols was also the same as in [38]: 5 s puff duration, 30 s interpuff lapse and 500 mL/min = 8.33 mL/s. Hence, our critique on [38] is directly applicable to this study (see our comments further ahead).

The authors analyzed (with GC-MS) 49 commercially available nicotine-free food grade flavor concentrates (β-damascone, δ-tetradecalactone, γ-decalactone, citral, dipentene, ethyl maltol, ethyl vanillin, linalool, and piperonal), as well as food grade ethyl vanillin propylene glycol acetal (found in more than 45% of flavors). Nearly 300 unique chemicals were identified in the concentrates. To examined thermal degradation of flavored aerosols, the concentrates were diluted to 20% into a mixture PG/VG 60:40 in similar concentrations found in commercial e-liquids.

Close to 43% of the flavors resulted in significant increases in radical production as compared to the base PG:GLY (60:40) mixture: from 46% (lemon) to 122% (vanilla custard), but significant reductions in radical production below baseline were observed as a result of adding Vanilla flavoring. The radical inhibition effects of ethyl vanillin suggest its possible use an additive in e-liquids reduce free radical production during aerosol formation.

Relative abundance of the different flavorants in each e-liquid concentrate was correlated with radical production. Strong correlations between found for β-damascone, δ-tetradecalactone, γ-decalactone, citral, dipentene, ethyl maltol, ethyl vanillin, ethyl vanillin PG acetal, linalool, and piperonal. Dipentene, ethyl maltol, citral, linalool, and piperonal promoted radical formation in a concentration-dependent manner. However, interestingly, ethyl vanillin inhibited the radical formation also in a concentration dependent manner. The capacity to oxidize biologically-relevant lipids was closely linked with free radical production.

To assess the results of this study we remark that all experiments were conducted with the tested EC device setup at 225 °C and 50 W, but aerosols were generated by a very low airflow, a rate 50% lower than that of the CORESTA 1 L/min airflow. This is important, since as we argued in the revision of [38] (and as shown in Figure 2 and discussed in Section 2) such a small airflow rate should produce a significant reduction of the power range of the optimal regime, rising the probability that the fixed 50 W used in the testing could be in the overheating region. Hence, it is not possible to rule out that the device was tested under overheating conditions and even a dry puff.

**Son et al. (2019)**. The authors [40] utilize the same experimental set up as in their 2020 study on carbonyls [21] and in a 2018 study [95] (see section), except that in the present study they only considered two power settings: 6.4 and 31.3 W. The main difference with other ROS studies is that they did not follow a nontargeted approach through EPR, but one targeting OH free radicals by the reaction of captured aerosol with disodium terephthalate (TPT) to form 2-hydroxyterephthalic acid (2OHTA), the known reaction product of OH and TPT. Their outcomes, given as nmol/puff of 2OHTA, are about ∼$10^{14}$–$10^{15}$ molecules/puff, an order of magnitude higher than those reported in [38,39,100]. However, this result is questionable, as it follows from the outcomes produced with a tested device puffed with a pure VG e-liquid, which is completely unrealistic in end users. Also, this large value occurs at maximal power of 31.3 W (see the high vertical box in the authors’ Figure 1). As we argue when reviewing the authors’ carbonyl study [21], it is very likely (on the grounds of their sample of vapers) that this maximal power level is an unrepresentative outlier (95 percentile). If the boxes for the pure VG e-liquid and this maximal power level are removed from their Figure 1, then 2OHTA levels become of magnitude $∼10^{14}$ like those found by EPR methods. However, the technique used by the authors is based on the 35% yield of the 2OHTA reaction that reflects the amount of OH radicals formed available for fluorescence detection. Letting all radical species from the degradation products remain in the reaction environment could give rise to further radical chains and lead to the formation of excess OH (through, for example, the Haber–Weiss reaction that produces hydroxyl radicals-OH from hydrogen peroxide-$H_{2}O_{2}$ and superoxide-$O_{2}$). This technique of quantifying OH free radicals might introduce uncertainties in OH quantification if not undertaken carefully (see details in [102]).

To estimate the exposure to OH radicals from ECs (from their outcomes) the authors assume the number of daily puffs for vapers to be in the range 10–1000. Taking 1000 puffs per day then they claim that OH exposure from ECs can be as high as (or higher than) that from tobacco cigarettes. Evidently, 1000 daily puffs is an extreme outlier that does not justify this alarming conclusion, besides the fact that their upper end outcome $10^{15}$ molecules/puff is an overestimation by an order of magnitud. Taking their representative values at 6.4 W, OH exposure from vaping is at most 1/100 of the exposure from smoking.

**Haddad et al. (2019)**. The authors [41] applied an optimized acellular 2’,7’-dichlorofluorescin (DCFH) probe technique to measure ROS in EC aerosols in sub-ohm and supra-ohm devices, varying power, e-liquid composition and nicotine concentrations. ROS emissions were quantified in the total particulate matter (TPM) of ECIG aerosols and tobacco smoke. For all device types ROS emissions were uncorrelated with power but were highly correlated with power per unit area. An increase in the e-liquid VG content produced higher ROS flux, but nicotine did not affect ROS emissions.

The devices used were: supra-ohm VaporFi platinum tank (5 and 11 W) and a sub-ohm Smok TFV8 device with a V8-T8 coil head (eight coils). Vaping sessions: 5 puffs (VaporFi platinum tank) and 2 puffs (Smok TFV8). Puffing protocol: 4-s puff duration, 10-s interpuff interval, 1 L/min flow rate for both EC devices and for cigarettes 10 puffs with ISO puffing protocol.

To assess the effects of power, the Smok TFV8 was vaped at 50, 75, 100, 150 and 200 W with a PG/VG 50:50 solution and 12 mg/mL nicotine concentration. The Smok TFV8 at fixed 50 W was used to assess the effect of different coil heads: V8-Q4 (4 coils), V8-T8 (8 coils), V8-T10 (10 coils) and TF-Q4 (4 coils). To examine the effects of e-liquid composition and nicotine concentration, the authors the following set up: PG/VG (100/0, 50/50 and 0/100 PG/VG) and (0, 6 and 12mg/mL) in a 50/50 PG/VG solution, all tested at 5 and 11 W (VaporFi platinum tank) and 50 and 150 W (Smok TFV8). Results are reported as the mean of three measurements after blank subtraction.

It is evident that the authors conducted the tests in this study under extreme and unrealistic conditions. A powerful sub-ohm device (Smok TFV8) is recommended for DTL vaping, but it is puffed with a CORESTA-like protocol with 1 L/min airflow and a short interpuff lapse (10 s) and at high powers 50, 75, 100, 150 and 200 W. Even if 50 W might be within the recommended power range of the manufacturers for DTL vaping, puffing this device at 1 L/min airflow narrows the power range of the optimal regime (see Section 2 and Figure 2) making it almost certain that the device was puffed under overheating conditions even at this lowest power used by the authors.

Regarding the VaporFi platinum tank, the authors mentioned it was activated by the aerosol lab vaping instrument (ALVIN) of The American University of Beirut, citing their reference (43) of a study the same author group published in 2015 [103], which describes such a device operating in the power range 3.0–7.5 W. However, the authors tested this instrument with the VaporFi tank at 5 and 11 W, which is above the operation power range, making it almost a certainty that at least some of the tests were conducted under dry puff conditions. Therefore, the experimental outcomes of these study for the sub-ohm Smok TFV8 device and the VaporFi platinum tank are completely irrelevant for end users.

## 9. Chemical Pathways of Solvents Degradation

We review in this section 5 studies whose main focus is not quantifying carbonyl byproducts in EC emissions, but improving the understanding of their production process from the chemical pathways of the thermal degradation of e-liquid solvents (PG and VG).

**Jensen, Strogin and Peyton (2017)**. This study [42] was not reviewed in [11], its main novelty and significance is its usage of spectral analysis from nuclear magnetic resonance (NMR) (instead of the usual analysis with LC MS or GC MS) to conduct an extensive probing of the chemical pathways of byproducts derived from thermal degradation of vaporized PG and VG. Compounds not previously detected were found in the pathway reactions with HCHO compounds, though only acetaldehyde and hydroxyacetone were quantified. The authors used a second generation Innokin iTaste VV4 with a KangerTech Protank-II claromizer, bottom coil 1.8–2.5 Ω and e-liquid PG/VG 50:50. Puff volume was kept fixed 55 mL, collecting 6–22 mg of aerosol by single puffs of varying duration: 3, 5 and 10 s. The wick was wetted and intervals between puff were 5 min or more as an attempt to avoid dry puffs and overheating.

However, varying puff duration with fixed puff volume leads for 5 and 10 s puffs to a very diminished airflow (11 and 5.5 mL/s). Even for such a low-powered device and without liquid depletion (verified by the authors), this puffing regime necessarily leads, as power increases 6–14 W, to overheating from insufficient evacuation of vaporized e-liquid and is also an artificial way to enhance carbonyl production (see large aldehyde outcomes for extended puff duration DP+ trials in [16]), besides the fact that 10 s puffs are extreme and unrealistic. The presence of overheating can be appreciated by the nonlinear relation obtained by plotting PG/VG generation (about >99.9% or aerosol mass) vs. power from the data in Table S1 of the Supplementary File. Evidently, the quantified acetaldehyde yields per puff for 10 s puffs are overexposures under abnormal conditions.

**Wang et al. (2017)**. The authors [43] examine the production of toxic volatile carbonyl compounds from PG and VG at varying a controlled temperature ranging up to 318 °C, by means of a “device-independent test method” utilizing a stainless steel, tubular reactor in flowing air that simulates a generic EC device.

The authors used e-liquids with pure PG and VG, a 50:50 PG/VG mix and two commercial ones. Acrolein was only detected in e-liquids containing VG and above 270 C. Formaldehyde and acetaldehyde were detected at reactor temperatures of 215 °C for both PG and VG, but at same temperature (318 °C) pure VG produced significantly higher yields: (in μg per mg of liquid): 21.1±3.80 vs. 2.03±0.80 (formaldehyde), 2.40±0.99 vs. 2.35±0.87 (acetaldehyde) and 0.80±0.50 vs. traces (acrolein). The authors claim that at 215 °C the estimated daily exposure to formaldehyde from e-cigarettes surpasses the USEPA and OEHHA toxicological thresholds.

The experimental set up is as follows: 5–10 mg of test e-liquid was loaded at the center of a 0.3 g piece of glass wool, which was then carefully transferred to the middle of a stainless steel, tubular reactor (25 cm long and 1 cm in inner diameter) housed in a horizontal, split-sided furnace. Contact between liquid and inner surface of the reactor was avoided. One end of the tube reactor was connected to compressed air with a regulated constant flow rate of 200 mL/min, to produce a 2.9 s transition time of e-liquid with air in the reactor to mimic a 3 s puff duration. Aerosols were collected onto DNPH cartridges and analyzed by HPLC.

The authors define as “reactor temperature” the temperature in the glass wool inside the reactor, fixed by a temperature controller and measured by a thermocouple. Although the test outcomes are shown in graphs at various fixed temperatures (215, 270, 315 °C), most likely these temperatures are reached by heating the reactor for a short time (the “transition time” mentioned before).

The displayed graphs for these fixed temperatures for 5 e-liquids show negligible yields of the 3 aldehydes below 215 °C, with yields rising at >215 °C (with larger increase for formaldehyde). Yields from VG are an order of magnitude larger than those of PG, which is in contradiction to results in other studies, a contradiction that the authors suggest can be explained by potential interaction of VG with reactor materials (stainless steel). The graphs for the PG/VG 50:50 mixture and a commercial e-liquid show the same qualitative behavior, but in the PG/VG mixture the rise of yields occurs below 215 °C and for the commercial liquid up to 250 °C.

This study is valuable for showing the temperature dependence of aldehyde production, even if it is evident that the tubular reactor used in this study is a poor simulator of an EC device (a fact the authors recognize). A steady flow of compressed air carrying e-liquid vapor at very low fixed airflow (3.33 mL/s) is an extremely crude inhalation model. As a consequence, the detected aldehyde yields cannot be extrapolated to those emerging from testing EC devices and the authors’ risk assessments are not relevant for end users.

Finally, the authors’ temperature measurements were not validated and thus do not seem to be reliable. However, even if temperature measurements were accurate, the temperature dependence of a process like aldehyde production is a thermodynamic process that cannot be modeled by temperature measurements from a sequence of fixed temperatures and a fixed airflow. We discuss this common conceptual error in Section 12.

**Li et al. (2020)**. The authors [44] present in detail a theoretical quantification model that can significantly contribute to a better understanding of the chemical pathways of carbonyl production in EC emissions.

Conventionally, carbonyl quantification is done by derivatizing with 2,4-dinitrophenyl hydrazine (2,4-DNPH) to produce hydrazone adducts, followed by LC or GC chromatography analysis with calibration of chromatographic peak areas guided by carbonyl-DNPH standards. Additional purification steps are required to isolate the mono and multi hydrazones in the DNPH synthesis. However, some carbonyls are not commercially available and further require a separate synthesis. The authors propose two goals: (1) to use high mass resolving power coupled to chromatography to better identify DNPH hydrazones of functionalized and simple carbonyls and acids, and (2) to develop a quantification for cases when analytical standards are unavailable.

To validate this technique the authors use a disposable device blu (Imperial Brands Inc., Bristol, United Kingdom), with non-refillable “Classic Tobacco” e-liquid cartridges. The device comprises a rechargeable 140 mA h battery and an atomizer with coil resistance of 3.5 Ω. Puffing protocol: two devices were puffed in tandem (4 puffs for each e-cigarette), puff duration 2 s with 8 puffs/min (7.5 s inter-puff lapse), average flow rate 2.3 L/min and puff volume 77 mL.

All analytes were separated in the chromatographic spectrum using accurate single ion cromatography, which once integrated into the full ion chromatography avoids coelusion and misidentification of spectral peaks that might lead to overestimation of abundance. A total of 19 DNPH hydrazones were identified: six hydroxycarbonyls, four dicarbonyls, three acids, and one phenolic carbonyl, with the most abbundant being hydroxyacetone, formaldehyde, acetaldehyde, lactaldehyde, acrolein, and dihydroxyacetone. As the authors remark, an important finding from this quantification method is that hydroxycarbonyls are just as important as simple carbonyls in the byproducts content of emissions.

The obtained carbonyl yields are displayed in their Table 2 (μg/puff): 0.15 formaldehyde, 0.11 acetaldehyde, 0.071 lactaldehyde, 0.061 acrolein, 0.037 dihydroxyacetone. By assuming 250 daily puffs they compute a daily formaldehyde exposure of 37.5 μg/day which is below the OEHHA daily exposure threshold of 180 μg/day. However, while the levels of mass abundances for the blu device were low, they are likely overestimations, as the authors’ puffing parameters are unrealistic: 2.3 L/min airflow and 77 mL puff volume are too high for a ciga-like (which has high air resistance), while at an 8 puffs/minute pace a full day vaping journey (250 puffs) would take only 31 min, an extremely exhaustive and intense form of vaping that bears no connection to real life usage.

**Melvin et al. (2020)**. This study [45] aims at the determination of the potential for the formation of α-dicarbonyl compounds, including diacetyl (DA), during the generation of aerosols from devices whose e-liquids contained no detectable DA or other α-dicarbonyl compounds. A model reaction system was set up to conduct mechanistic studies using a model microwave reaction system to identify key reaction precursors for DA. The same reference e-liquid (50:50 PG/VG + 2.5% (*w*/*w*) nicotine + 15% water (*w*/*w*)) from the method validation study was used for the subsequent evaluations of reactant combinations. The increase in DA content between the native e-liquid and the aerosol in all tested devices was indicative of its formation during aerosol generation potentially through a thermal degradation pathway.

The 8 commercial ciga-like devices that were tested were acquired in 2017 at local convenience stores, all contained rechargeable batteries with disposable e-liquid cartridges. However, the authors only identify two devices obtained internally: MarkTen XL Classic and MarkTen XL Menthol (products A and B, respectively). The puffing protocol was 55 mL puff volume, 5 s puff duration, and 30 s puff interval. The aerosol was collected using the standard methods, and 25 puffs were collected from each sample.

Concentrations of DA in the aerosols of the 8 tested tested devices were about 20–40 times lower than established occupational DA exposure limits of the American Conference of Governmental Industrial Hygienists (ACGIH): 0.04 mg/$m^{3}$ (0.01 ppm) and the recommended exposure limit (REL) National Institute of Occupational Safety and Health (NIOSH): 0.02 mg/$m^{3}$ (0.005 ppm). These outcomes lead to a daily exposure of 1/360 with respect to daily DA exposure from tobacco cigarette smoke (250 μg/cigarette).

The main shortcoming of this study is its choice of tested devices: old design ciga-likes of which the only two that were identified are of the Mark10 brand that is currently obsolete or of very marginal usage. Also, the study was published in 2020, 3 years after the acquisition of the devices in 2017, so that corrosion or leaching effects cannot be ruled out, as no information was supplied on their storage conditions. While the authors correctly claim that these results only apply to the ciga-likes they tested, these old design devices release higher levels of byproducts than more modern devices [11].

**Jaegers et al. (2021**) The authors [46] simulate an EC device by means of a single cavity using an in situ MAS NMR rotor containing e-liquid, gas, and metal solid samples (${\mathrm{ZrO}}_{2}$ and ${\mathrm{Cr}}_{2}O_{3}$) to simulate coil materials. The rotor is transferred to a specially designed loading chamber where $N_{2}$ or $O_{2}$ is added at the specified pressures together with e-liquids to mimic the quantity of vapor evolved in an average puff (∼10–50 mg). The purpose of the experiment is to monitor the transition of e-liquids in order to identify the decomposition products that might be present at low-temperatures (<200 °C) in EC emission pathways by controlling the oxygen availability and the temperature.

However, monitoring the detection of converted chemical species by natural abundance ${}^{13}C$ and ${}^{1}H$ NMR in the suggested low-temperature degradation pathway requires an extended time period (the MAS NMR spectra takes days to generate peaks at same temperature). Under the authors’ experimental conditions, if sufficient oxygen is available, e-liquids liquids decompose at temperatures <200 °C forming byproducts (formic and acrylic acids) via an oxygen initiated radical-mediated mechanism.

While this study can be a valuable contribution to enhance current knowledge on the chemistry of degradation paths in EC emissions, its experimental setup bears no relation with the physics of EC operation. A loading chamber with the required oxygen supply necessary for the days long monitoring of e-liquid transitions at fixed target temperatures below 200 °C is a closed and highly controlled system, whereas an EC device is an open thermal system, with rapidly time and space varying temperatures and turbulent air fluxes (oxygen supply) as it is exchanging heat between its parts and with the environment, a large thermal bath (see Section 12). It is highly likely that these physical conditions might prevent the reaction pathways found by the authors to take place in EC devices, but this should be assessed by further experimental research.

## 10. Carbonyls and Nicotine Compensatory Behavior

We review two studies by the same group of researchers that illustrate how reduction of nicotine levels leads to a compensatory behavior characterized by an increase of device power and/or more intense puffing. These studies show that such compensatory behavior significantly increases the emission of toxic byproducts, in particular formaldehyde and acetaldehyde.

**Dawkins et al. (2018)**. This observational study [47] was conducted during 4 weeks between September 2016 and February 2017, as a follow up of a previous study by the same group of researchers [76]. It is based on ad libitum vaping of 20 experienced vapers who received for the duration of the study the same device: an eVic Supreme (Joytech) endowed with adjustable voltage and recording the time for each puff, puff length (in seconds), atomizer resistance (1.6 Ω fixed), voltage and wattage. The eVic was fitted to a Nautilus Aspire tank. Participants received e-liquids with the two mentioned nicotine levels choosing flavors they sampled beforehand (tobacco, menthol, fruit and bakery).

This sample of users was used to examine the compensatory effects of nicotine levels and variability of power settings in a combination of 4 setups given by: high/low nicotine level (18 vs. 6 mg/mL) each with adjustable/fixed power settings, spending one week observation for each combination and looking at the interaction between them. The authors measured in this sample puffing parameters (daily puffs, puff duration, interpuff interval), e-liquid consumption, power settings (when not fixed), subjective effects (urge to vape), as well as metabolite biomarkers of nicotine (salivary cotinine), acrolein and formaldehyde (urinary 3-Hydroxypropyl mercapturic acid, 3-HPMA and formate). The study corroborates and provides further observational support to a previous pilot study (by same group) that examined compensatory effects in puffing habits when decreasing nicotine concentration in e-liquids (with analogous compensatory behavior in cigarette smoking).

The results displayed in the authors’ Table 3 reveal an increase of about 12–18% in mean daily puffs for lower nicotine levels respect higher ones: 338 vs. 279 (fixed power) and 308 vs. 279 (variable power). Mean puff duration increased 20% from high to low nicotine levels (3.61 to 4.46 s), but only for fixed power, but e-liquid consumption increased for lower nicotine levels, 17% and 26% for variable and fixed power respectively. High nicotine levels decreased withdrawal symptoms. However, these compensatory effects on low nicotine concentrations are not sufficient to maintain a stable nicotine intake, as cotinine levels were higher in high nicotine 18 mg/mL concentration irrespective of fixed/variable power setting. While acrolein metabolite levels were insensitive to the setup combinations, formaldehyde metabolites showed a significant increase in lower nicotine levels in both power settings.

**Kosmider et al. (2020)**. This study [48] is the upgrade and continuation of the previous study by the same research group [47], following a similar methodology and using exactly the same EC device, but now conducting laboratory tests whose puffing protocol was taken from the average puffing parameters observed in 19 experienced vapers in the 4 combination of: e-liquids with low/high nicotine levels (6 and 18 mg/mL) and fixed/variable power adjustments. The laboratory tests quantified daily exposure to carbonyl emissions by HPLC. The results further confirm those of [47]: significantly higher e-liquid consumption and thus higher exposure to formaldehyde and acetaldehyde were found when switching from higher to lower nicotine levels, regardless of power settings.

Table 2 of the study reveals statistically significant higher levels of daily nicotine intake for e-liquids with higher nicotine concentrations (mean ± SD, mg/day): 22.69±15.16 vs. 13.22±8.93 (fixed power) and 27.87±16.86 vs. 15.23±10.49 (adjustable power). However, low nicotine e-liquids produced significantly higher daily levels of aerosol yield (median, range, g/day): 2.26 (1.46–4.22) vs. 1.38 (0.7–3.01) (fixed power) and 2.64 (1.71–4.85) vs. 1.38 (0.7–3.01) (adjustable power); formaldehyde dose (μg/day, median range): 26.83 (11.99–56.11) vs. 13.69 (6.95–27.99) (fixed power) and 25.63 (15.82–70.74) vs. 20.23 (10.1–45.57) (adjustable power); acetaldehyde dose 19.91 (4.68–66.47) vs. 8.18 (3.42–34.03) (fixed power) and 20.17 (5.61–83.00) vs. 8.18 (3.42–34.03) (adjustable power). Following the methodology of Stephens, the authors also showed that the compensatory behavior of low nicotine levels contributes to a 1.98 fold increase (2.06 and 1.26 fold with fixed and adjustable voltage) of the Cancer Risk Index (CRO) associated with formaldehyde and acetaldehyde. The authors’ assessments roughly agrees with the assessment of Stephens model estimating CRI from EC usage being 3–4 orders of magnitude below cigarette smoking.

## 11. Summary and Evaluation

On the grounds of the quality criteria presented in Section 4, we provide our evaluation of the revised studies listed in Table 1 in terms of the graphic codes displayed in Table 2: experimental consistency and reproducibility are graded with tickmark symbols: ✔, **+/−**, ✖, **?** and toxicological confidence with a “traffic light” system ●, ●, ●, ●. Table 3 and Table 4 display how each study rates according to these codes. This is a broad general evaluation, details are discussed extensively in the individual revision of each study on Section 6, Section 7, Section 8, Section 9 and Section 10 (Author names in the left columns of Table 3 and Table 4 are hyperlinked to their review entries in these sections).

**Table 2:** **Evaluation Codes.** Toxicological Confidence (traphic lights) and Experimental Consistency (tickmark symbols).

Toxicological Confidence			
●	●	●	●
Fully Reliable	Restricted Reliability	Completely Unreliable	Missing Information
**Experimental Consistency**			
✔	**+/−**	✖	**?**
Fully Consistent	Restricted Consistency	Completely Inconsistent	Missing Information

A quick recount of the evaluation provided in Table 3 and Table 4 reveals a significant pattern in 35 revised studies that quantified organic byproducts. The most frequent drawbacks were those marked as (1) and (2) in the “Comments” column in the extreme right of Table 3 and Table 4: testing sub-ohm devices with a CORESTA or CORESTA-like protocol. This was found in 18 out of 35 studies, with:

- 10 testing them at too high power levels, thus almost certain overheating and most likely irrelevant to all end users (graded as ✖)
- 8 under recommended power levels, thus with likelihood of overheating with very restricted relevance limited to a small minority of users, see Section 2, Section 3.1 and Section 12.1, (graded as **+/−**).

Regarding the traffic light toxicological confidence grading, 12 rated as unreliable (●), 12 as very restricted reliabilty (●), 11 as reliable (●), with a strong correlation between gradings ●/● and causes (1), (2). Therefore, this general evaluation clearly reveals frequent inappropriate testing of sub-ohm devices, with likely overestimation of health risks from these experiments, thus suggesting also the necessity to upgrade laboratory standards to address this frequent problem.

**Table 3:** **Studies on carbonyls 2018–2022**. Author names are hyperlinked to review entries in Section 6. The tickmark symbols and traffic light codes are given in Table 2: [✔, **+/−**, ✖, **?**] and [●, ●, ●, ●] are respectively [Fully Consistent, Restricted Consistency, Completely Inconsistent, Missing information] and [Fully Reliable, Restricted Reliability, Completely Unreliable, Missing information]. The number codes in the column “Comments” are: (1) sub-ohm device with CORESTA high powers, (2) sub-ohm device with CORESTA recommended powers, (3) other forms of inconsistent protocol, (4) incorrect computation of exposure, (5) outliers not properly identified, (6) devices not fully identified, (7) testing power not identified, (8) too frequent puffs, (9) too long puffs, (10) used old devices.

	Experimental Consistency		Reproducibility		Toxicological Confidence	Comments
First Author & Hyperlink	Vaping Regime	Power Range	Emissions Generation	Trapping & Analysis		
CARBONYLS						
2018						
Vreeke [13]	**+/−**	**+/−**	✔	✔	●	(1)
Korzun [14]	✔	✔	✔	✔	●	(4)
El Hellani [15]	**+/−**	✔	✔	✔	●	(4)(5)(8)
2019						
Beauval [16]	**+/−**	**+/−**	✔	✔	●	(2)
Ooi [17]	✖	✖	**?**	**?**	●	(1)(3)(4)(5)(6)
Balushkin [18]	**+/−**	**+/−**	✔	✔	●	(2)(10)
Reilly [19]	✔	✔	✔	✔	●	
2020						
Son [21]	✔	**+/−**	✔	✔	●	(5)
Talih [22]	✖	**+/−**	✔	✔	●	(1)(5)
Zelinkova [23]	✖	✔	✔	✔	●	(2)
Uchiyama [24]	✖	✖	✔	✖	●	(1)(2)(6)
Cunningham [25]	✔	✔	✔	✔	●	
Noël [26]	✖	✖	**?**	✔	●	(1)(4)(6)
Mallock [27]	✔	✔	✔	✔	●	
2021						
Chen [28]	✔	✔	✔	✔	●	
Crosswhite [29]	✔	✔	✔	✔	●	
Li [30]	**+/−**	✔	✔	✔	●	(2)(3)(4)
Yan [31]	✖	✖	✔	✔	●	(1)(3)(4)
Tehrani [32]	✖	✖	✔	✔	●	(1)(3)(4)(7)(8)
Cancelada [20]	**+/−**	**+/−**	✔	✔	●	(2)(3)
2022						
Xu [33]	✔	✔	✔	✔	●	
Visser [34]	✔	✔	✔	✔	●	

**Table 4:** **Studies on CO, ROS, degradation pathways and nicotine vs. carbonyls**. Author names are hyperlinked to review entries in Section 7, Section 8, Section 9 and Section 10. The traffic light codes are given in Table 2: [✔, **+/−**, ✖, **?**] and [●, ●, ●, ●] are respectively [Fully Consistent, Restricted Consistency, Completely Inconsistent, Missing information] and [Fully Reliable, Restricted Reliability, Completely Unreliable, Missing information]. The number codes in the column “Comments” are: (1) sub-ohm device with CORESTA high powers, (2) sub-ohm device with CORESTA recommended powers, (3) other forms of inconsistent protocol, (4) incorrect computation of exposure, (5) outliers not properly identified, (6) devices not fully identified, (7) testing power not identified, (8) too frequent puffs, (9) too long puffs, (10) used old devices. (NA) stands for “does not apply” (these studies only simulated an EC).

	Experimental Consistency		Reproducibility		Toxicological Confidence	Comments
First Author & Hyperlink	Vaping Regime	Power Range	Emissions Generation	Trapping & Analysis		
CO						
Casebolt [35]	✖	✖	**+/−**	✔	●	(1)(3)
El Hellani [36]	✖	✖	**+/1**	✔	●	(1)(3)(4)
Son [37]	**+/−**	✔	✔	✔	●	(2)(3)
ROS						
Bitzer (a) [38]	✖	**+/−**	✔	✔	●	(2)(3)
Bitzer (b) [39]	✖	✖	✔	✔	●	(2)(3)
Son [40]	✔	**+/−**	✔	✔	●	(3)(4)
Haddad [41]	✖	✖	**+/−**	✔	●	(1)(3)(9)
Degradation reactions & carbonyl formation						
Jensen [42]	✔	✔	✔	✔	●	(9)
Wang [43]	(NA)	**+/−**	**+/−**	✔	●	(3)
Li [44]	✔	✔	✔	✔	●	(8)(9)
Melvin [45]	✔	✔	**+/−**	✔	●	(6)(9)(10)
Jaegers [46]	(NA)	(NA)	✔	✔	(NA)	
Nicotine compensation vs. carbonyls						
Dawkins [47]	✔	✔	✔	✔	●	
Kosmider [48]	✔	✔	✔	✔	●	

## 12. Discussion

### 12.1. Testing Sub-Ohm Devices with Insufficient Airflow

Since the CORESTA protocol used in most of the revised literature was conceived for testing early ciga-like devices, its puffing parameters (airflow 1 L/min, puff volume 50–70 mL) are appropriate for testing the low powered recent device types (cartridge based and refillable pods, disposables) used by substantial numbers of vapers for MTL style, specially young adults [60,62,63]. The CORESTA or CORESTA-like puffing parameters might not be wholly appropriate for testing even those devices that are also meant for MTL vaping, but operate at power levels 10–30 W above recent pods and disposables. As shown in [49], it is very likely that these “intermediate” devices are fully compatible with airflows of up to 4 L/min larger than those specified by CORESTA or CORESTA-like protocols.

However, it is certain that puff volumes and airflows of CORESTA or CORESTA-like protocols are very problematic for testing sub-ohm devices that are mostly used for DTL vaping, as this vaping style involves much larger puff volumes and airflows (see Section 3.1 and Section 3.2). As we show in Section 6, Section 7 and Section 8 and summarized in Section 11, at least half (18 out of 35) of laboratory emission studies have tested sub-ohm devices with CORESTA or CORESTA-like protocols. Out of these 18 studies, 10 tested the devices also at too high powers levels [13,17,22,24,26,31,32,35,36,41], hence under almost certain overheating, which renders their outcomes and risk assessments from sub-ohm devices completely irrelevant for end users. The remaining 8 studies tested the devices at recommended power levels [16,18,20,23,24,30,37,38,39], hence overheating is possible but not certain. Evidently, only these latter 8 studies provide outcomes and risk assessments from sub-ohm devices that might be relevant for at least a minority niche of vapers. We estimate in this section that this minority constitutes around 5% of all vapers, roughly the proportion of vapers using high powered sub-ohm devices with MTL style.

As stated in Section 3.1, there is abundant anecdotal accounts of a very strong correlation between vaping styles and devices type from vaping forums and magazines, consumers and retailers. This correlation was also observed in the footage material examined in [54]: 80% of users of sub-ohm devices practice DTL and 98% of DTL vapers use sub-ohm devices, while 95% of users of low-powered supra-ohm devices practice MTL. Interestingly, the 20% minority of MTL vapers using sub-ohm devices take on average shorter puffs than the 80% DTL majority. Given the absence of insufficiently documented evidence, we can assume as a plausible working hypothesis the observed data in [54], with a vast majority (likely 80%) of high powered sub-ohm devices used for DTL vaping, with a minority of users (likely 20% or less) using them for the MTL style. thus, it is likely that outcomes of studies testing sub-ohm devices with CORESTA or CORESTA-like protocols might be relevant and realistic for this minority niche (which might not be a stable niche).

Since there is no direct demographic evidence, the distribution of MTL and DTL styles can be inferred indirectly (and very approximately) from demographic surveys in the US (2018–2020) [59], the UK (2022) [60] and Germany (2018) [61] that have inquired the type of device among vapers, showing that between 50–65% use “refillable tank models”, with the rest using cartridge based and refillable pods. While all DTL vaping will necessarily correspond to tank models, not all tank models are sub-ohm devices meant for DTL. Since, as we argued in Section 3.1, usage of sub-ohm devices involves more expertise and maintenance than low powered devices, so it should occur typically among long time ex-smoking vapers. Since between 40–60% of vapers are now ex-smokers in the US and UK markets [59,60], it is reasonable to assume (as a working hypothesis) that sub-ohm devices are used by a large minority within the 40–60% of ex-smokers, which should roughly translate into 20%-25% of all vapers, with 75–80% using low powered devices (though demographic trends seem to show a steady evolution to low powered devices and even disposables [60,62,63]).

If the relevance of outcomes for testing sub-ohm devices with CORESTA or CORESTA-like protocols is restricted to those practicing MTL style with these devices. To estimate the proportion of this minority niche among all vapers, we assume as working hypothesis that (i) ∼25% of vapers use sub-ohm devices (likely an overestimation) and (ii) the observational data in [54] stating that ∼80% of these vapers practice DTL and ∼20% MTL. The proportion of vapers practicing MTL style in sub-ohm devices is then roughly ∼5% of all vapers (∼20% of ∼25%). Hence, outcomes and health risk assessments from testing sub-ohm devices with CORESTA or CORESTA-like protocols should be directly useful and relevant to a really small minority of vapers. Evidently, we have produced a very rough estimation of usage sub-ohm devices for MTL style, but even if this minority habit combination is larger than 5%, it is evident that these outcomes and risk assessments are not applicable to the substantial numbers of vapers using low powered devices (we discuss this issue further in Section 13).

### 12.2. Arbitrarily Fixing Power, Temperature and Puff Duration

Some of the revised studie considered ad hoc combination of varying/fixed parameters. Son et al. [37] varied power with fixed puff volume at 40 mL and airflow with fixed power at 50 Watts, Li et al. [30] varied the coil temperature under a CORESTA-like protocol with fixed airflow 1.186 LT/min and puff volume 55 mL. Cancelada et al. [20] varied airflow (opening the inlet venting holes in the atomizer) with fixed power, while Bitzer et al. [38] varied temperature with fixed power and power with fixed temperature, but at a fixed CORESTA-like airflow. While these studies present interesting results, their set of combinations of puffing parameters are disconnected with those of the vast majority of consumers, as usage of sub-ohm devices typically involves increasing puff volumes and airflows together with increasing power and temperature. Nevertheless, as shown in Table S9 of Son et al, the reported emitted mass of CO and all aldehydes are of the order of ng/puff for all tested puff flows, which is about 1 part in $10^{7}$ of the aerosol mass of 84±29.6 mg/puff (which should be close to the consumed e-liquid mass).

The fist problem with fixing the temperature is the lack of information on how it is evaluated by the device instrumentation. The second problem is the lack of experimental validation of temperature values, an important shortcoming because aerosol generation and vaping involve complex patterns of heat conduction that (in general) produce a time varying and inhomogeneous temperature. If the end user can determine a prior a fixed power or temperature to vape, this necessarily requires the user to control (i.e., to vary in a sequence of trials) the intensity of the inhalation (airflow, puff volume, puff duration) to levels that are adequate according to sensorial and taste criteria. In technical terms this process involves finding the appropriate puffing parameters (airflow, puff volume, puff duration) that allow for an efficient condensation (by forced convection in inhalation) of vaporized e-liquid that forms the aerosol in the optimal regime. Besides being wholly artificial and disconnected with realistic usage, testing sub-ohm devices with fixed airflows and puff volumes of CORESTA or CORESTA-like protocols for different (but fixed) power and temperature are not likely adequate for carrying on this condensation process efficiently and will more likely fail to comply with users’ sensorial and good taste criteria (see Section 2 and Section 3).

### 12.3. Physical Limitations on Chemical Processes

Chemical reactions can be studied and recreated in a wide range of temperatures that can be controlled in a laboratory, thus allowing for a better understanding of the different steps of degradation pathways. This approach can hardly be applied to a dynamic environment of an EC device in which it is extremely difficult to regulate correctly the temperature, which is not an extensive state variable that can be determined, fixed and supplied externally.

A chemical approach in a laboratory is very difficult to implement in an EC device, thus it is conceptually incorrect to present potential degradation reactions under the assumption that they will occur if testing an EC device by setting the temperature at a desired value from the device instruments. This conceptual error leads to the assumption that as power increases it leads proportionally to a more rapid evolution of the vaporization process through a higher heating temperature. In reality, e-liquid vaporization occurs through physical phenomena involving several regimes and limits which results in the ignition of different chemical degradations, it cannot be produced or induced by arbitrarily fixing the temperature to increasing constant values. This criticism applies to Li et al. [30], Bitzer et al. [38], Wang et al. [43] and Jaegers et al. [46].

The first main assumption is that an EC device is functional between specific powers (minimal and maximal powers) limiting an optimal regime of vaporization characterized by to nucleated boiling. Through this regime, e-liquid consumption linearly increases with icreasing the supplied power. However, these limits are obviously influenced by e-liquid composition and also by the vaping regime and to find them requires a consistent experimental verification.

The second main assumptions is a thermodynamic conceptual issue. Vaping products are open systems ventilated by fresh air induced by inhalation. This leads to the mixture of a gas at ambient conditions (pressure, moisture and temperature) to another formed by the vaporization of e-liquid (effects that cannot be reproduced in the simulated devices in [43,46]). It results in an aerosol at a temperature intermediate between the temperatures of each separated gas. Therefore, e-liquid constituents cannot be heated at temperatures above, at least, the boiling temperature of pure glycerol. Considering conditions above this assumption leads to experiments under overheating conditions.

Saliba et al. [104] in 2018 investigated the pyrolysis of PG using a quartz pyrolyric reaction in the presence and absence of a metallic coil available in vaping devices. The reactor was heated at 80, 256, 360, 460, 565 and 670 °C. With the exception of 80 °C, the remaining temperature conditions are above the boiling temperature of PG, which allows for exploring PG degradation in the gas phase. This is a typical example of an inappropriate in-lab experiment extrapolated to a vaping device. Indeed, PG has a boiling temperature of 188 °C. However, in a liquid solution, interactions with others compounds as glycerol can maintain PG in liquid phase at higher temperature as 256 °C. Therefore, only the conditions at 80 °C can be extrapolated to normal conditions of use of a vaping device.

These two fundamental assumptions highlight another chemically originated inconsistent approach. Some studies (Wang et al. [43] and Jaegers et al. [46]) conducted experiments with e-liquids in closed systems (an oven, a linear flow reactor) that allow for the heating of a controlled volume of a gas and/or a liquid sample. For many physical considerations, this type of experiments can lead to contradictory results or overestimation of risks from excess byproduct formation, but are irrelevant to the study of EC devices because of their incompatibility with the functionality of these devices as thermodynamically open systems.

## 13. Assessment of Risk Communication

Since EC emissions contain HPHCs, one of the main tasks of their testing in the laboratory is to provide objective guidelines to assess health risks that end users (and bystanders) would face if exposed to specific doses of these emissions. Most revised studies present assessments of health risks based on their experimental quantification of the abundance of potentially toxic byproducts, an abundance that must be compared with compound-specific toxicological markers. A substantial proportion of the risk assessments express serious concerns, some cast doubts on the safety of the devices and even recommend harsh regulatory restrictions [17,26,31,32].

However, the extensive revision of laboratory studies we have conducted shows that most of them have failed (in various degrees) to uphold the basic consistency criteria we described in Section 4. Therefore, we recommend due care and skepticism in evaluating most risk assessments, but question the objectivity of the most severe ones. We have evaluated the toxicological reliability of the risk assessments in 35 revised studies by a traffic light system in Table 3 and Table 4 [●, ●, ●, ●], repectively corresponding to [reliable, limited reliability, unreliable, missing information].

From the extensive literature revision carried on in Section 6 to Section 10, summarized in Table 2, Table 3 and Table 4 of Section 11, the most frequent form of inconsistency is testing sub-ohm devices under CORESTA or CORESTA-like puffing protocols: 18 of 35 studies, marked with with (1) and (2) in the Comments column of Table 3 and Table 4. From the outset, on the grounds of arguments of Section 2 and Section 3, we can unequivocally state that risk assessments from these studies are:

- questionable and unrealistic for all users if coming from the outcomes of 10 studies that used high power settings with these protocols to test sub-ohm devices [17,22,24,26,31,32,35,36,41]. The 10 studies are marked with red traffic light ● in Table 3 and Table 4. It is practically certain that these testing conditions lead to overheating and possibly dry puffs, likely producing aerosols that could be repellent to end users (see Section 3.3).
- of very limited validity if coming from 8 studies that used these protocols to test sub-ohm devices, but at recommended power settings [13,16,18,20,23,24,30,37,38,39]. These assessments must be taken with skepticism, as they come from testing conditions that might involve overheating as a likelihood, but not as a near certainty (the corresponding traffic light is ●). As we argue in Section 3.1 and Section 12.1, risk assessments from these testing conditions with recommended power settings are only relevant to a minority of consumers using these devices with an MTL vaping style, likely ∼20% of users of these devices and ∼5% of all users (since users of sub-ohm devices are very likely a minority of vapers).
- completely unaplicable and irrelevant for low powered pod and disposable devices used by a substantial proportion of vapers (at least 35–40% of vapers according to demographic surveys [59,60,61,62,63]).

Other problems in the experimental design (see Comments column of Table 3 and Table 4) affect the reliability of risk assessments. Some studies miscalculated (overestimated) exposure in various ways [14,17,26,30,31,32,36,40]. Some risk assessment are based on ranges of outcomes that contain unrepresentative outlier values [15,21,37,40]. A number of studies failed to disclose important information to assess their results and open the possibility of replication [17,24,26,32,45]. Some studies tested devices under excessive puffing frequency [15,32,44]: multiple puffs one every 10 s or less, when demographic studies of vaping behavior (see Section 3.4) reveal that such bouts of up to 20 rapid puffs only occurs for short time periods, but is not representative of daily usage. Two studies tested old ciga-like devices and devices acquired years before testing without describing storage conditions [18,45], all of which makes it impossible to rule out corrosion or leaching effects. It is interesting to remark that the same inconsistencies were found in our review of laboratory studies that focused on metal contents in EC aerosol emissions [5].

The most frequent and abundant HPHCs reported in the revised literature are aldehydes, specially formaldehyde, acetaldehyde and acrolein, all formed from reactions of thermal degradation of the e-liquid solvents and whose toxic potential is well known. Less is known of the inhalation toxicity from the lesser yields of intermediate byproducts of these reactions, which as shown in [30,44] might play as important a role in PG and VG degradation as primary carbonyls. Free radicals, specially hydroxyl OHs, are also concerning. However, all HPHCs were found in negligible dose in comparison with tobacco smoke in all studies testing low powered devices without gross inconsistencies in their experimental design (as for example [17,32]). Evidently, the incorporation of vapers in the logistics of testing procedures would contribute to improve the objectivity of risks assessments, as suggested in the important review [11], something that (among all revised studies) only [21,40] attempted with a limited scope, but [34,53] have taken the first steps in a more thorough implementation.

## 14. Conclusions

We have undertaken in this review an extensive critical revision of 36 laboratory studies published since 2018 on organic byproducts (carbonyls, CO and free radicals) in EC emissions. Details of the experiments, our criticism of their design and toxicological considerations are written in the extensive reviews in Section 6, Section 7, Section 8, Section 9 and Section 10 and summarized in Table 1, Table 2 and Table 3 in Section 11. This summary is complemented by a thorough critical discussion on multiple aspects of the laboratory studies in Section 12. As in our previous review on metal contents [5], we have focused primarily on the consistency between aerosol generation by puffing protocols in the laboratory and an efficient aerosol generation fulfilling the physical constraints of the optimal regime (Section 2) in the tested devices and validated when they are operated by their end users, whose vaping habits and sensorial perceptions are discussed in detail in Section 3.

One of the main purposes of laboratory testing of emissions is to provide evidence of potential health risks from exposure to HPHCs in EC emissions. To fulfill this purpose efficiently, the studies must comply with minimal consistency conditions that we describe in Section 4. We evaluated in Section 11 these consistency conditions for all revised studies by a system of tick marks (experimental consistency) and traffic lights (toxicological confidence). As we stated in [5], we question the objectivity of negative health risk assessments and harsh policy recommendations that emerge from studies undertaken under severe experimental inconsistencies, including clear evident overheating conditions (rated with ✖ and ● in Table 2 and Table 3). Studies with less severe inconsistencies, marked by **+/−** and ● in Table 2 and Table 3, require to be carefully examined under a skeptical approach.

The revision of the literature we have undertaken further supports the available empiric evidence from self-consistent laboratory studies that vaping is a much safer option of nicotine consumption than smoking, thus motivating the support of its role in tobacco harm reduction. Nevertheless, EC emissions still involve residual health risks from inhalation and exposure to HPHC’s, even if self consistent experiments find them in minute quantities in comparison with their presence in tobacco smoke. Long term risks (even if residual) are difficult to forecast, model and predict and the analysis and quantification of HPHCs in EC emissions is only the front line of a complex process of subsequent risk assessments to forecast, probe and test the biological and medical effects of the inhaled chemicals through studies of biomarkers, cytotoxicity, animal models, preclinical and clinical studies. However, for risk assessments from emission studies to be a useful component of this process and be of utility to stakeholders (users, bystanders, health professionals, manufacturers and regulators), these assessments must be solidly grounded on HPHC outcomes that have been quantified in well designed self consistent experiments testing the devices under conditions that best approach their realistic usage, otherwise these risk assessments can be fictitious and irrelevant.

Besides being the front line in estimating health risks from vaping, laboratory testing of emissions is essential for quality control, product comparison and technological development. While it is not expected that its standardized regimented procedures will reproduce real life user patterns, at least a minimal consistency with realistic usage must hold for the testing to have any relevance for end users. As in our previous review on metal studies, we have found in the literature on organic byproducts that most studies fail (in various degrees) to uphold this consistency, some studies often fail to do so even at the most basic levels.

The main methodological problem that we found (in 18 of 35 studies) is testing high powered “sub-ohm” devices whose aerosol emissions are generated with the puffing parameters of the CORESTA protocol (and/or with minor deviations), a protocol conceived and designed to be appropriate for testing early low-powered devices. Testing sub-ohm devices with these protocols is very likely unrealistic, as these devices are mostly used for the ’Direct to Lung’ (DTL) style involving much higher airflows and puff volumes. Other serious methodological problems are: (i) failure to disclose important information on the devices, the puffing parameters, testing power ranges, the outcomes, making interpretation of results more difficult and future replication impossible; (ii) misleading health risk assessments, either from taking outlier outcomes as representative or simply miscalculation of exposures; (iii) testing old devices without disclosing their storage conditions and current status, (iv) excessively frequent puffing and/or too long duration puffs.

These methodological problems are not new, most were identified already in 2018 in the groundbreaking review by Farsalinos and Gillman [11] of 32 emissions studies published up to 2017 on carbonyls (see Section 5), which mostly tested fist and second generation low-powered ECs available at the time. After 5 years, with so many newer devices emerging constantly into the market, we found very similar inconsistencies in this review (see Section 11) and in our recently published review of studies focused on metal contents in EC emissions [5]. Looking back, it seems that the technological development of ECs has evolved much more rapidly than the methods and standards used to analyze the device emissions.

As we mentioned in [5] and after further extending the arguments in Section 2, the main root of these methodological problems stems from the lack of understanding of the thermal physics involved in aerosol formation in a vaping device, based on the equilibrium of various heat exchanges (specially between e-liquid vaporization by supplied power and forced convection to form aerosol by condensation), an equilibrium that leads (when broken) to abnormal overheating conditions and a dry puff phenomenon with pyrolysis of the wick. Understanding these phenomena makes it possible to determine in the laboratory (for each device and e-liquid) the experimental settings compatible with the physical conditions (power ranges, coils, e-liquids) that allow for vaping to proceed efficiently in an optimal regime according to this equilibrium.

As we explain in Section 2, testing powerful sub-ohm devices under the insufficient airflow and puff volumes of the CORESTA or CORESTA-like protocols (the most frequent problem we have found) reduces the power ranges for an optimal regime. Pending on the testing power ranges, this leads to either a higher likelihood or a certainty of overheating conditions (see Section 3.1 and Section 12.1). Surprisingly, 10 of 18 revised studies that used CORESTA or CORESTA-like protocols to test these devices did so at high power settings above the optimal regime, under clear overheating conditions. Evidently, outcomes and risk assessments from these studies are irrelevant for end users. The remaining 8 studies tested the devices at power ranges within recommended values, leading to a high likelihood (but not certainty) of overheating conditions. However, as we show in Section 12.1 and Section 13, even if avoiding overheating, the experimental outcomes and risk assessments from these 8 studies are only relevant for a small minority (∼20%) of users of these devices with the lower airflows of the ’Mouth to Lung’ (MTL) vaping style, likely ∼5% of users in general. The outcomes and risk assessments of the 18 studies testing sub-ohm devices with CORESTA or CORESTA-like protocols are likely irrelevant for ∼95% of vapers (given the substantial proportion using low powered devices).

We also express again the urgent need to update and upgrade the current CORESTA testing standard, a standard created in the early days of vaping as an adaptation of the puffing protocols of tobacco cigarettes to the old ciga-likes that resembled them, but very insufficient for testing the wide variety of currently available devices. As shown in Section 2, probing the parameters of the optimal regime (power settings and mass of e-liquid vaporized) for specific devices and e-liquids, should be a requirement prior to performing laboratory testing under conditions that avoid overheating.

Most of the studies we revised have conducted experiments without or with very insufficient input from the habits and needs of consumers and the evolution and trends in the market of vaping products. As pointed out and emphasized by Farsalinos and Gillman in [11], it should be a priority to complement laboratory testing by incorporating recruited vapers to assist in the logistics of experiments, as end users can perceive sensorially the effects (hot aerosol, foul taste) associated with a break down of an efficient vaping. It is also necessary to study in more detail how vapers vape, for example conducting research focused on

- Creating a data base of optimal regime laboratory testing for large samples of devices, e-liquids and coils, as described in Section 2 and [8,9]. This data basis can contribute to identify power ranges that avoid overheating.
- Cohort longitudinal studies for updating the scientific knowledge on the puffing behavior of EC users for all currently available devices.
- The development of inbuilt safety features, as user alerting systems, on EC devices that will at least notify users that the device is operating beyond its optimal regime with thermodynamic efficiency.
- Guidelines to inform and regulate the market of old age EC devices and their maintenance and storage conditions.

The present review shows that laboratory testing requires a much more flexible standard, not only providing appropriate technical guidelines, but facilitating the incorporation of end users to complement laboratory logistics. As future research we are planing an experimental replication of various emission studies that we revised in this review and in [5].

## Figures and Tables

**Table 1:** Classification by subject of studies under review.

Reviewed Studies	Section	References
2 previous review articles	Section 5	[11,12]
22 studies on carbonyls and byproducts	Section 6	[13,14,15,16,17,18,19,20,21,22,23,24,25,26,27,28,29,30,31,32,33,34]
3 studies on CO	Section 7	[35,36,37]
4 studies on ROS	Section 8	[38,39,40,41]
5 studies on byproduct formation	Section 9	[42,43,44,45,46]
2 studies on carbonyls vs. nicotine	Section 10	[47,48]

## Data Availability

Not applicable.

## Abbreviations

The following abbreviations are broadly used in this manuscript (other abbreviations are fully described where defined):

EC	Electronic Cigarette
HPHC	Harmful and Potentially Harmful Compounds
CO	Carbon Monoxide
ROS	Reactive Oxygen Species
CORESTA	Cooperation Centre for Scientific Research Relative to Tobacco
CORESTA-like	Minor modifications of CORESTA protocols
MTL	Mouth to Lung
DTL	Direct to Lung
MEV	Mass of E-liquid Vaporized
PG	Propylene glycol
VG	Vegetable glycerine (glycerol)
DNPH	2,4-dinitrophenyl hydrazine (2,4-DNPH)
LC	Liquid Chromatography
GC	Gas Chromatography
MS	Mass Spectrometry
HP	High Performance
HR	High Resolution
UV	Ultraviolet
EPR	Electron Paramagnetic Resonance
NMR	Nuclear Magnetic Resonance
ICP	Inductively Coupled Plasma
C	degrees centigrades
pg	picogram
ng	nanogram
μg	microgram
g	gram
mg	milligram
mL	milliliter
L	Litter
cm	centimeter
m	meter
s	secondsr
min	minutes
h	hour
W	watts
V	Volts
Ω	ohms
